# Robust and efficient knock-in in embryonic stem cells and early-stage embryos of the common marmoset using the CRISPR-Cas9 system

**DOI:** 10.1038/s41598-018-37990-w

**Published:** 2019-02-06

**Authors:** Sho Yoshimatsu, Junko Okahara, Takefumi Sone, Yuta Takeda, Mari Nakamura, Erika Sasaki, Noriyuki Kishi, Seiji Shiozawa, Hideyuki Okano

**Affiliations:** 1 Department of Physiology, School of Medicine, Keio University, Shinjuku-ku, Tokyo 160-8582 Japan; 2 Laboratory for Proteolytic Neuroscience, RIKEN Center for Brain Science, Wako City, Saitama 351-0198 Japan; 3 Laboratory for Marmoset Neural Architecture, RIKEN Center for Brain Science, Wako City, Saitama 351-0198 Japan; 4 Central Institute for Experimental Animals, Kawasaki, Kanagawa 210-0821 Japan

## Abstract

Genome editing technology greatly facilitates the genetic modification of various cells and animals. The common marmoset (*Callithrix jacchus*), a small non-human primate which exhibits high reproductive efficiency, is a widely used animal model in biomedical research. Developing genome editing techniques in the common marmoset will further enhance its utility. Here, we report the successful establishment of a knock-in (KI) method for marmoset embryonic stem cells (ESCs), which is based on the CRISPR-Cas9 system. The use of CRISPR-Cas9, mediated by homologous recombination (HR), enhanced the KI efficiency in marmoset ESCs. Furthermore, we succeeded in performing KI in early-stage marmoset embryos. In the course of the experiments, we found that HR in the marmoset ESCs is innately highly efficient. This suggested that the marmoset possesses a repair mechanism for DNA double-strand breaks. The current study will facilitate the generation of genetically modified marmosets and gene function analysis in the marmoset.

## Introduction

There are two major pathways for repairing DNA double-strand breaks (DSBs)^1–3^. One is non-homologous end joining (NHEJ), which is an error-prone, but dominant pathway for repairing DSB in mammalian cells^2^. The other is homologous recombination (HR), an error-free pathway for DSB repair that employs a chromatid or exogenous DNA as the template^3^. The choice of the repair pathway depends on the species, cell type, and cell cycle^1^.

Gene targeting using HR, namely knock-in (KI) and knock-out (KO), is widely used for disease modelling and gene function analysis. In particular, the KI strategy is utilized for multiple applications, such as the introduction of a gene-specific reporter or a missense mutation at a specific region of the genomic DNA. However, in mammalian cells, the frequency of HR is low compared to that of random integration which is mediated by NHEJ^1^. Thus, in these cells, homologous recombinants are more difficult to obtain than randomly integrated clones^4,5^.

Targeted endonucleases, such as zinc-finger nuclease (ZFN), transcription activator-like effector nuclease (TALEN), and clustered regularly interspaced palindromic repeats (CRISPR)-Cas9 are used as genome editing tools for NHEJ-mediated gene KO^6^. In addition, since HR is triggered by DSB^7^, targeted endonucleases have been used for canonical gene targeting to increase the efficiency of HR. This approach is highly effective and enables genetic modification via HR, not only in embryonic stem cells (ESCs) of various species, but also in early-stage embryos^6^. Out of these technologies, the CRISPR-Cas9 system is particularly convenient, since the *streptococcus pyogenes* Cas9 endonuclease only requires 3-bp PAM sequence and a chimeric single guide RNA (gRNA) that is complementary to the target genomic sequence (20 nucleotides) to induce DSB at a specific genomic locus^8^.

The common marmoset (*Callithrix jacchus*) is highly useful as a non-human primate biomedical model, because of its high fertility, short gestation period (144 days), and high physiological similarity to humans^9,10^. Our group has previously reported the successful generation of transgenic and KO marmosets^11,12^ and demonstrated the utility of marmoset models for studying diseases. However, the methods to generate such models are still under development and should be improved to enable more efficient generation. Here, we present a HR-mediated gene targeting method combined with the CRISPR-Cas9 genome editing technique in marmoset ESCs (cjESCs). Using this method, we obtained high KI efficiencies in several different genetic loci of cjESCs. Furthermore, we used this method to successfully perform KI in early-stage marmoset embryos. Surprisingly, not only did we establish a method to obtain high KI efficiency in cjESCs, but also, we found that cjESCs possess innate high HR activity. Especially, in the case of targeting exon 1 in the *proteolipid protein 1* (*PLP1*) gene, almost all of the clones that survived positive selection were homologous recombinants, even without using CRISPR-Cas9. This unique feature will be useful for future studies in disease modelling and gene function analysis in marmosets, and possibly other non-human primates.

## Results

### Evaluation of KI efficiency in cjESCs using the CRISPR-Cas9 system

To test whether the CRISPR-Cas9 system works in marmoset cells, we introduced marmoset-specific gRNA sequences (summarized in Supplementary Table 2) into pSpCas9-2A-Puro (Cas9-gRNA vector; PX459) and evaluated the genomic cleavage activity (GCA) of Cas9 and the gRNAs in cjESCs by transfecting each Cas9-gRNA vector and transiently selecting the transfected cjESCs with puromycin (see Experimental procedures). We confirmed the GCA of Cas9 and all of the gRNAs to be used in the current study (Supplementary Fig. S1a–h).

**Figure 1:**
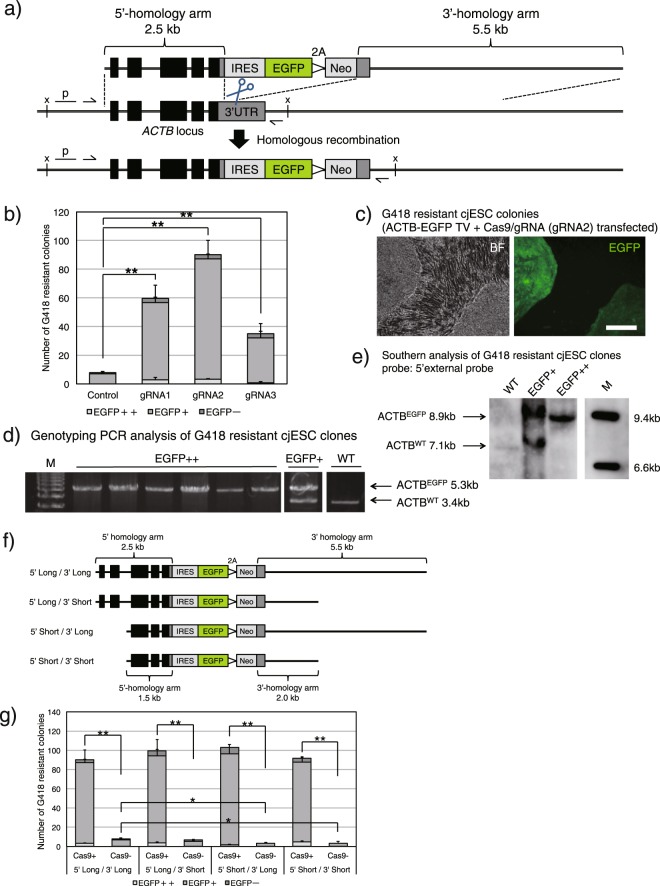
CRISPR-Cas9 enhances KI efficiency in cjESCs. (**a**) Schematic diagram of the ACTB-EGFP system. The *ACTB-EGFP* TV harboured *IRES-EGFP-2A-Neo* flanked by 2.5-kb and 5.5-kb homology arms to the surrounding regions of the 3′-UTR of the marmoset *ACTB* gene locus. The three gRNAs (ACTB-1, 2, 3, their recognition sites are shown as scissors) target the 3′-UTR region, which is not included in the TV. The TV is not detected by the gRNAs for the marmoset *ACTB* gene. Black thin arrows show the primer binding sites for genotyping PCR; x, a restriction enzyme site (*XbaI*); p, 5′-external probe for Southern blotting. (**b**) The number of G418-resistant colonies following G418 selection of 1 × 10^6^ transfected ESCs, shown as the mean + s.e.m., n = 3. The number of colonies with strong EGFP fluorescence (EGFP++) is shown in dark grey; the number of colonies with moderate EGFP fluorescence (EGFP+) is shown in grey; the number of EGFP-negative colonies (EGFP−) is shown in bright grey. (**c**) A representative image of an EGFP++ (left) and EGFP+ (right) colony observed under bright field (BF) or under green fluorescence after G418 selection. Scale bar, 200 μm. (**d**) Genotyping PCR analysis of EGFP++ and EGFP+ cjESC clones. M, DNA marker. The separate images were cropped from the same gel. (**e**) Southern blotting analysis of EGFP++ and EGFP+ clones using the 5′-external probe. M, DNA marker. The separate images were cropped from the same gel. The entire image of the gel is shown in Supplementary Fig. S14a. (**f**) Schematic diagram of the shortened *ACTB-EGFP* TVs. (**g**) The number of G418-resistant colonies following selection of 1 × 10^6^ transfected cjESCs, shown as the mean + s.e.m., n = 3. Each group is represented by the same colours as in (**b**). *P < 0.05, **P < 0.01.

Next, we decided to target the *ACTB* gene locus to test whether CRISPR-Cas9 enhances KI efficiency in cjESCs. Previously, by using a promoter-trapping *ACTB-EGFP* targeting vector (TV) carrying a G418 resistance gene (Fig. 1a)^13^, we confirmed that most of the G418-resistant and EGFP-positive colonies are homologous recombinants. Therefore, the number of G418-resistant and EGFP-positive colonies are considered to be indexes for KI efficiency.

For preparation of the experiment, we first designed three gRNAs (ACTB-1, 2, and 3; Supplementary Table 2) targeting the 3′-UTR of the marmoset *ACTB* gene and transfected the cjESCs with the *ACTB-EGFP* TV, with or without each corresponding Cas9-gRNA vector. The numbers of EGFP-positive and -negative colonies were counted following positive selection with G418 for two weeks. After transfection of 1 × 10^6^ cjESCs with or without each Cas9-gRNA vector, we found that the numbers of G418-resistant colonies significantly increased in the Cas9-gRNA transfected cultures (Cas9-gRNA(+)) (gRNA1: 59.6 ± 14.9, gRNA2: 90.0 ± 14.4, gRNA3: 34.9 ± 12.3; Fig. 1b), compared to that in the non-transfected control cultures (Cas9-gRNA(−)) (control: 8.0 ± 1.2; Fig. 1b). In addition, in the Cas9 gRNA(+) group, we noticed that some EGFP-positive colonies showed an apparently stronger EGFP fluorescence (the left colony in Fig. 1c) than others (the right colony in Fig. 1c). Therefore, we cloned six EGFP-positive colonies with high EGFP fluorescence (EGFP++) and one colony with moderate EGFP fluorescence (EGFP+). Genotyping analysis of these clones revealed that all of the EGFP++ clones were homozygous recombinants (Fig. 1d–e) without any additional TV integrations (Supplementary Fig. S2), while the EGFP+ clone was a heterozygous recombinant (Fig. 1d,e). These observations indicated that CRISPR-Cas9 genome editing increased KI efficiency in cjESCs.

We next evaluated the KI efficiency using three newly constructed *ACTB-EGFP* TVs with shortened homology arms (Fig. 1f). As expected, using the shortened TVs resulted in the reduction of KI efficiency in the control group that was not transfected with Cas9-gRNA. However, we did not see a decrease in KI efficiency when Cas9-gRNA (gRNA2) was transfected (Fig. 1g).

In addition, in order to estimate the KI efficiency without having to perform positive selection, we also evaluated the transfection efficiency and colony formation efficiency immediately following transfection. Transfection with a *mVenus* expression vector (pCXN2-mVenus) revealed that the transfection efficiency was 32.0 ± 6.3% (n = 5), and colonies were formed from 1.97 ± 0.26% (n = 4) of passaged cjESCs. Thus, from 1 × 10^6^ cjESCs, approximately 6300 colonies were transfected and expected to form colonies before positive selection. Accordingly, in the gRNA2 and control group, the targeting efficiency of transfected colony-forming cjESCs was calculated to be approximately 1.43% (gRNA2) and 0.13% (control). To experimentally validate this approximate calculation, we performed fluorescent-activated cell sorting (FACS) analysis. In short, we transfected cjESCs with the *ACTB-EGFP* TV and Cas9-gRNA vector (gRNA2), and transiently selected the cells with puromycin. These cjESCs were further expanded, and the proportion of EGFP-positive (EGFP(+)) cells was analyzed by FACS. The PX459 alone was used as the control. In the control group (gRNA(−)), there were few EGFP(+) cells, calculated to be around 0.18 ± 0.05% (Supplementary Fig. S3a). In the gRNA2 group (gRNA(+)), the percentage of EGFP(+) cells were increased to 1.75 ± 0.17% (Supplementary Fig. S3b), which was a significant increase when compared to the control (*P* < 0.001; Supplementary Fig. S3c). This result indicates that the number of counted cjESC colonies which underwent positive selection reflects KI efficiency, and the approximate calculation obtained using the *mVenus* expression vector helps to translate the number of counted colonies into KI efficiency to some extent.

### Evaluation of KI efficiency in a non-expressed gene in cjESCs

**Figure 2:**
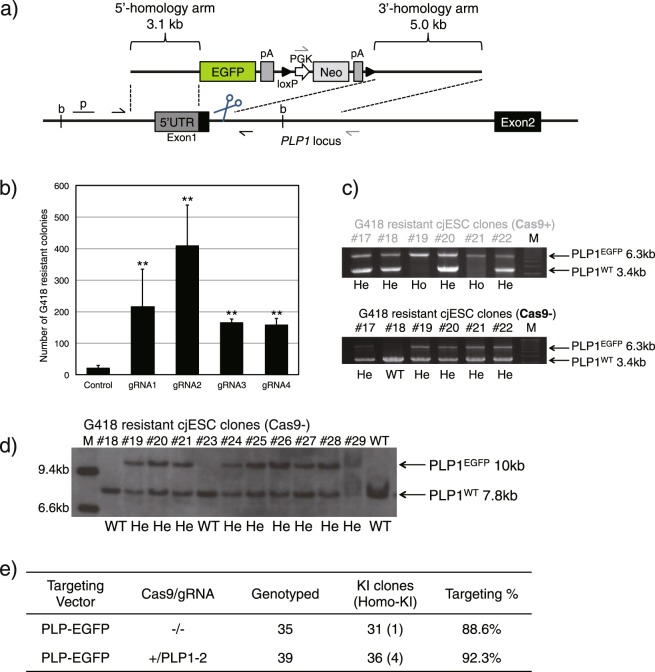
Evaluation of KI efficiency for an inactive gene. (**a**) Schematic diagram of the *PLP1-EGFP* construct. The *PLP1-EGFP* TV harboured 3.1-kb and 5.0-kb homology arms, which consisted of the region surrounding the initial codon of the marmoset *PLP1* gene locus. EGFP coding sequence was fused to the 3′ end of the 5′-UTR of the *PLP1* gene. Polyadenylation signal sequence (*pA*) was introduced downstream of the terminal codon of *EGFP*. Additionally, loxP-flanked *PGK-Neo-pA* was placed between *EGFP-pA* and the 3′-homology arm. The TV was not detected by gRNAs targeting the vicinity of *PLP1* exon 1 (PLP-1, 2, 3, 4, shown as scissors). Thin black arrows indicate the location of the 5′-external primer and the 3′-internal primer used for genotyping PCR of the 5′-region. Grey arrows indicate the 5′-primer homologous to the selection cassette and the 3′-external primer used for genotyping PCR of the 3′-region; b, a restriction enzyme site (*Bgl*II); p, the 5′-external probe for Southern blotting. (**b**) The number of G418-resistant colonies following G418 selection of 1 × 10^6^ transfected cjESCs, shown as the mean + s.e.m., n = 3. (**c**) 5′-region genotyping PCR of G418-resistant cjESC clones. Cas9+ clone numbers are shown in grey (#17–22), and Cas9− are shown in black (#17–22). Results for the remaining clones are shown in Supplementary Fig. S4a. Ho, homozygous KI; He, heterozygous KI. (**d**) Southern blotting analysis of G418-resistant Cas9− clones using the 5′-external probe. (**e**) Summary of the *PLP1* exon 1 gene targeting data. *P < 0.05, **P < 0.01.

We demonstrated the impact of genome editing through targeting of the *ACTB* gene with a promoter-trap strategy and found that CRISPR-Cas9 indeed increased the number of homologous recombinants. Next, we tested a non-promoter trap strategy at the *PLP1* gene locus, which is normally not expressed in cjESCs. PLP1 is a transmembrane proteolipid protein abundantly expressed in oligodendrocytes (OLs)^13^. Deletion or mutation of the encoding gene causes Pelizaeus-Merzbacher disease (PMD) and spastic paraplegia 2^14^. We constructed four gRNAs targeting different regions which were all in the vicinity of *PLP1* exon 1 (PLP1-1, 2, 3, 4; Supplementary Table 2) and a *PLP1-EGFP* TV, which carries the loxP-flanked *PGK-Neo* cassette to target the initiation codon of *PLP1* exon 1 (Fig. 2a). We transfected cjESCs with the *PLP1-EGFP* TV, with each Cas9-gRNA vector or without (control). When the Cas9-gRNA vectors were used, the numbers of colonies that appeared following the G418 selection increased approximately 8–20 fold (gRNA1: 215.0 ± 119.0, gRNA2: 408.5 ± 128.8, gRNA3: 163.5 ± 12.5, gRNA4: 156.5 ± 22.5, n = 3; Fig. 2b), compared to the control (20.0 ± 9.1, n = 3; Fig. 2b). Because most colonies obtained using the non-promoter trap strategy are usually randomly integrated clones, it was intriguing that the total number of colonies that were homologously recombined were increased when transfecting Cas9-gRNA. We cloned 39 G418-resistant colonies from cjESCs transfected with both the TV and the Cas9-gRNA (gRNA: PLP1-2) vector (Cas9+ clones), and 35 from cjESCs transfected with only TV (Cas9− clones). Genotyping polymerase chain reaction (PCR) revealed that 4/39 (10.3%) Cas9+ clones were homozygous, and 34/39 (87.2%) Cas9+ clones were heterozygous KI clones (Fig. 2c and Supplementary Fig. S4a). Surprisingly, even among the Cas9− clones, 1/35 (2.9%) was homozygous and 31/35 (88.6%) were heterozygous KI clones. This revealed an unexpectedly high KI efficiency with and even without Cas9-gRNA in the G418-resistant cjESC clones. Genotyping PCR was also conducted for the 3′-region of the targeted region; 37/39 (94.9%) Cas9+ clones and 32/35 (91.4%) Cas9− clones were correctly targeted (Supplementary Fig. S4b). In addition, eleven Cas9− clones were subjected to Southern blotting, which confirmed that homologous recombination occurred at the correct locus (Fig. 2d). The genotyping data for G418-resistant clones are summarized in Fig. 2e. Furthermore, to demonstrate the utility of the *PLP1-EGFP* KI reporter, we differentiated one homozygous-KI cjESC clone (Cas9− #11) into neuronal cells including OLs following excising the loxP-flanked *PGK-Neo* cassette (Supplementary Fig. S5a–e). Although we failed to detect EGFP fluorescence of the KI cjESCs in an undifferentiated state (Supplementary Fig. S5d, left), EGFP-positive cells emerged from the neurosphere stage (Supplementary Fig. S5d, center). Overall, <5% cells were EGFP-positive among the total differentiated cell on day 70, and at the time point 50–60% of EGFP-positive cells were co-stained with mature OL markers (MBP and GalC; Supplementary Fig. S5f).

In order to verify that a high ratio of KI clones was obtained from G418-resistant Cas9− cjESC clones (Fig. 2e), we also performed genotyping PCR using bulk genomic DNA extracted from the G418-resistant colonies from each well which was transfected with *PLP1-EGFP* TV, either with or without the Cas9-gRNA (gRNA: PLP1-2; Cas9(+) or Cas9(−) wells). As a result, the KI band intensities, which was normalized to the WT band intensity, were marginally lower in the Cas9(−) wells (36.9%, 34.1%) than those in the Cas9(+) wells (37.2%, 48.0%) (Supplementary Fig. S6a).

Furthermore, to further verify that a sampling or differentiation bias did not influence the KI efficiency of cjESCs, we performed quantitative reverse transcription-PCR (qRT-PCR) analysis of pluripotency-related genes *OCT4* and *NANOG*. Results showed that the cjESCs maintained a similar undifferentiated state either with or without G418 selection, which indicates that the high KI ratio was not due to a differentiation bias of the cjESCs during G418 selection (Supplementary Fig. S6b). In addition, the efficient KI of *PLP1* exon 1 without using Cas9-gRNA was confirmed in another cjESC line (Supplementary Fig. S6c). These observations suggested an innate high HR activity in cjESCs.

Next, we attempted to introduce pathogenic missense mutations in exons 5, 6, and 2 of the *PLP1* gene^15,16^. When TV constructs, *PLP1*-P216S or *PLP1*-S253T targeting *PLP1* exons 5 or 6, respectively (Supplementary Fig. S7a) were transfected, CRISPR-Cas9 increased the number of G418-resistant colonies (Supplementary Fig. S7b) and the KI efficiencies (Supplementary Figs S7c,d and S9). Precise KI in the correct locus was confirmed by DNA sequencing in multiple clones, but some clones were found to harbour a mosaic or WT sequence in the KI allele (Supplementary Fig. S8a,b, and Table 5). Likewise, in the case of transfecting the *PLP1*-A39T construct into *PLP1* exon 2 (Supplementary Fig. S7e), the number of puromycin-resistant colonies (Supplementary Fig. S7f) and KI efficiencies increased (Supplementary Figs S7g and S9). There were no abnormal sequences in the KI clones transfected with gRNA2 and gRNA3 (PLP1-CDS2-2 and PLP1-CDS2-3, respectively), while some mosaicism was found in clones transfected with gRNA1 and gRNA4 (PLP1-CDS2-1 and PLP1-CDS2-4, respectively) clones (Supplementary Fig. S8c and Supplementary Table 5). These results are summarized in Supplementary Fig. S7h.

### Humanization of the *FOXP2* gene

Since *PLP1* is an X-chromosomal gene, we next tested our method in FOXP2, an autosomal gene. *Forkhead box protein P2 (FOXP2)* gene is a transcription factor related to lung and corticobasal development^17,18^. In addition, several evolutional and pathogenic mutations in the gene are associated with the language ability of humans^19,20^.

**Figure 3:**
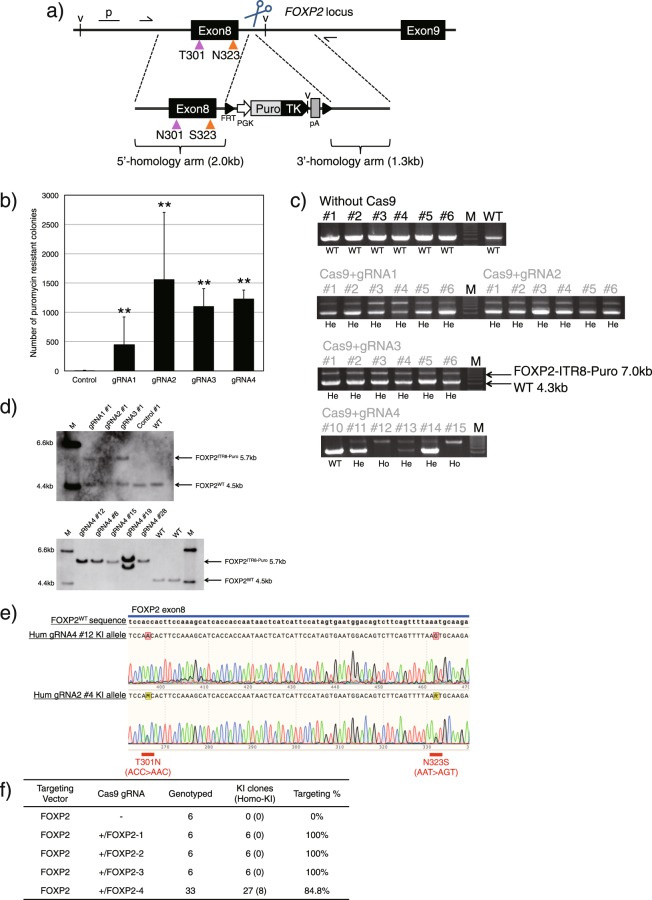
Humanization of the *FOXP2* gene. (**a**) Schematic diagram of *FOXP2* targeting. The FOXP2 TV harboured FRT-flanked *PGK-PuroTK-pA* cassette between the 2.0-kb and 1.3-kb homology arms for intron 8 of the *FOXP2* gene locus. The magenta and orange arrowheads indicate two human-specific substitutions, T301N and N323S, respectively; the sequences were introduced to the *FOXP2* exon 8 locus of the TV. The TV was not detected by gRNAs for the marmoset *FOXP2* gene (FOXP2-1, 2, 3, 4, shown as scissors). Thin black arrows indicate the 5′- and 3′-external primers used for genotyping PCR; v, a restriction enzyme site (*Eco*RV); p, the 5′-external probe for Southern blotting. (**b**) The number of puromycin-resistant colonies following selection of 1 × 10^6^ transfected cjESCs, shown as the mean + s.e.m., n = 3. (**c**) Genotyping PCR analysis of the puromycin-resistant ESC clones (*FOXP2* humanization). Among the clones of gRNA4, six representative clones (#11–15) are shown, and the remaining clones are shown in Supplementary Fig. S10. (**d**) Southern blotting analysis of puromycin-resistant cjESC clones using the 5′-external probe. The data of eight clones (gRNA1 #1, gRNA2 #1, gRNA3 #1, control #1, gRNA4 #12, 8, 15, 28) genotyped by Southern blotting matched that of genotyping PCR, except for one aberrantly recombinated clone (gRNA4 #19). (**e**) DNA sequencing analysis of the KI alleles. A representative mutated KI clone (gRNA4 #12) and a mosaic clone (gRNA2 #4) are shown. (**f**) Summary of the *FOXP2* gene targeting data. *P < 0.05, **P < 0.01.

We constructed three gRNAs for *FOXP2* intron 8 and one for exon 8 (FOXP2-1, 2, 3, 4, respectively; Supplementary Table 2) and a *FOXP2* TV, harbouring sequences for two human-specific amino acids (T301N and N323S)^20^ in *FOXP2* exon 8 and a *PGK-PuroTK-pA* cassette flanked by FLP recognition target sites (FRT)^21^ (Fig. 3a). We transfected cjESCs with the TV, either with or without each Cas9-gRNA vector. The use of CRISPR-Cas9 increased the number of puromycin-resistant colonies by 55-191 times (Fig. 3b). Furthermore, 0/6 (0%) of Cas9− clones, 6/6 (100%) of Cas9+ gRNA1, 6/6 (100%) of Cas9+ gRNA2, 6/6 (100%) of Cas9+ gRNA3, and 28/33 (84.8%) of Cas9+ gRNA4 clones were KI clones (Fig. 3c). Additionally, transfection with Cas9 and gRNA4 resulted in the efficient generation of homozygous KI clones (8/33, 24.2%; Fig. 3c and Supplementary Fig. S10). Eight clones were also genotyped by Southern blotting. The resulting data matched that of the genotyping PCR, except for one clone (gRNA4 #19) in which aberrant recombination occurred (Fig. 3d). However, when using Cas9 and gRNAs targeting intron 8 (FOXP2-1, 2, 3), DNA sequencing analysis of the KI allele band from PCR analysis (7.0 kb) revealed that the sequences of exon 8 in the KI alleles were WT or mosaic (mixed WT and humanized sequences; Fig. 3e, bottom, and Supplementary Table 5). On the other hand, KI clones generated by transfecting cjESCs with the TV and Cas9-gRNA targeting exon 8 (FOXP2-4) had humanized sequences in exon 8 in all of the KI alleles (Fig. 3e, top, and Supplementary Table 5). The genotyping data of the puromycin-resistant clones are shown in Fig. 3f.

In conclusion, by using the *FOXP2* TV and Cas9-gRNA (gRNA: FOXP2-4) vector, we were able to successfully generate homozygous KI clones harbouring human-specific mutations at the correct site.

### Evaluation of KI efficiency in early-stage marmoset embryo

**Figure 4:**
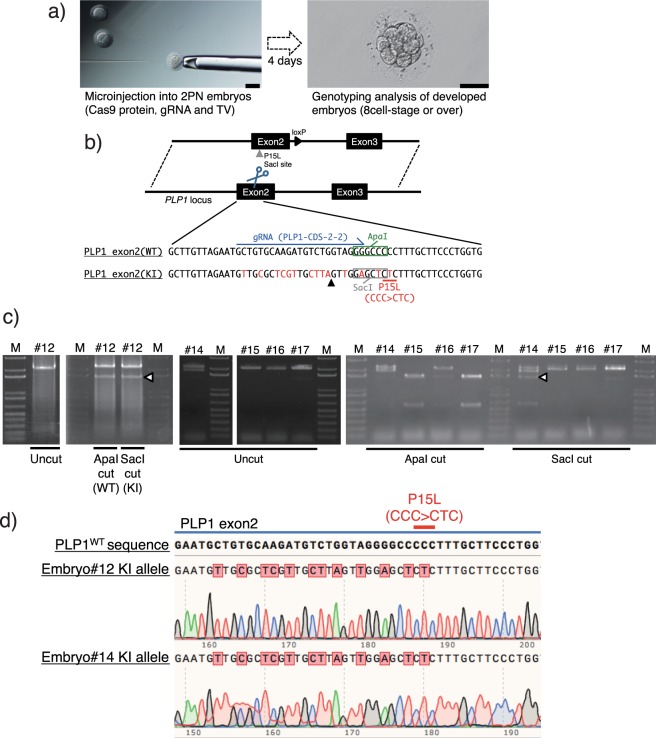
Evaluation of KI efficiency in the early-stage marmoset embryo. (**a**) Schematic of the gene targeting experiment using early-stage marmoset embryos. 2PN, the two-pronuclear stage. Scale bar, 50 μm. (**b**) Schematic of the *PLP1*-P15L KI construct in the embryos. Grey arrowhead indicates the location of sequence resulting in the P15L substitution and the *Sac*I site in the TV. Scissors indicate the DSB site. Thin blue arrow indicates the gRNA-targeted sequence (including the PAM sequence of *streptococcus pyogenes Cas9*; NGG). Black arrowhead indicates the Cas9-cleaved sequence site. (**c**) RFLP analysis of the microinjected embryos (#12 and #14–17, the remaining results are shown in Supplementary Fig. S12b). White arrowheads indicate detection of the KI allele by *Sac*I digestion of the PCR fragments. (**d**) DNA sequence analysis of the KI allele of embryos #12 and #14. Results for the remaining embryos are appended in Supplementary Fig. S12c.

We have demonstrated that the developed KI method for cjESCs resulted in high KI efficiency. Next, we attempted to apply this KI method to early-stage marmoset embryos (Fig. 4a). For this experiment, we selected the *PLP1* exon 2 KI construct (Fig. 4b), since this construct enabled homologous recombination in cjESCs the most efficiently (Supplementary Figs S7e–h, S8c, S9, and Table 5) among the experiments described above. In addition, the *PLP1*-CDS2-2 gRNA induced KI the most efficiently among the four gRNAs targeting *PLP1* exon 2 (Supplementary Fig. S7f) with the least concern of mosaicism in cjESCs (Supplementary Table 5), so we used the gRNA for further experiments with early-stage embryos. We used the *PLP1*-P15L TV encoding the pathogenic P15L substitution^22^ for *PLP1* exon 2 and harbouring a *Sac*I restriction enzyme site instead of an *Apa*I restriction enzyme site in the WT allele (Fig. 4b). We performed genotyping PCR using the entire genomic DNA extracted from each embryo which developed to the 8-cell stage or beyond (Fig. 4a, right). The PCR products were subjected to restriction fragment-length polymorphism (RFLP) analysis and DNA sequencing after subcloning the product into a blunt vector. The RFLP analysis of the KI using the TV was first validated in cjESCs (Fig. S11a and b).

Based on preliminary experiments (data not shown), we optimized the concentration of each component in the microinjection solution. The optimized solution consisted of the Cas9 protein (100 ng/μl), annealed crRNA and tracrRNA (50 ng/μl), and TV (100 ng/μl) in nuclease-free water, which were injected into the cytoplasm of two pronuclear-stage (2PN) zygotes (Fig. 4a, left). For the microinjection, 38 embryos were used; 18/38 (48.6%) embryos developed to the 8-cell stage or beyond; and 17 of them were genotyped. Surprisingly, under this condition, all the genotyped embryos were genetically modified (Fig. S12a and c). In 4/17 (24%) embryos (#2, #9, #12, #14), the PCR fragment was digested by *Sac*I, which confirmed KI (Fig. 4c, Supplementary S12b). KI was further validated with DNA sequencing of the subcloned PCR fragments (Fig. 4d and Supplementary Fig. S12c). At least 7 subclones per embryo were analyzed. However, more than two sequence variations were found in 7/17 (41%) embryos, which showed that mosaicism occurred in these embryos (Supplementary Fig S12c). These results are summarized in Supplementary Fig. S12a.

Furthermore, we utilized a zygote-electroporation method for early-stage marmoset embryos. Referring to previous studies performed in mice embryos^23,24^, we set an electroporation condition for marmoset embryos. Components of the electroporation solution included the Cas9 protein (100 ng/μl), annealed crRNA and tracrRNA (50 ng/μl), and the TV (100 ng/μl) in 1x OPTI-MEM. Additionally, since successful KI using a double-stranded DNA vector has never been achieved by electroporation in rodent embryos^23–25^, we utilized a 200-bp single-stranded oligonucleotide (ssODN) which overlaps the DSB site of the PLP1-CDS2-2 gRNA, and encodes the P15L substitution, *Sac*I site, and silent mutations, included for identifying the type of KI template transfected in each embryo. The ssODN-mediated KI was validated in cjESCs by RFLP analysis. The ssODN showed lower KI efficiency (2.8% and 4.1%) compared to that with the TV (3.9% and 8.3%) in our condition (n = 2; Supplementary Fig. S11b), different from a previous report showing an increased efficiency in KI for ssODN relative to TV^26^ but consistent with a previous study using murine ESCs^27^, However, further experiments would be required to make a definitive conclusion on this subject. The sequence of the ssODN is appended in the Supplementary Information. For these experiments, 7 embryos were used, 6 (86%) of them developed to the 8-cell stage or beyond, and subsequently genotyped. We found that 4/6 (66%) embryos carried genetically modified alleles, and 2/6 (33%) embryos had KI alleles resulting from transfection of either the ssODN or the TV, which were detected by DNA sequencing and RFLP analysis (Supplementary Fig. S13b and c). The data are summarized in Supplementary Fig. S13a.

Thus, by using the microinjection and electroporation conditions we developed and optimized, we succeeded in efficiently introducing gene modifications in marmoset embryos, resulting in either KI or KO.

## Discussion

In the current study, we established a gene targeting method for cjESCs and early-stage embryos, using CRISPR-Cas9 for directed DSB. This method dramatically increased the number of colonies that survived positive selection, and enabled bi-allelic homologous recombination. Furthermore, this method is robust, since KI of several genetic loci, including genes that are normally not expressed in undifferentiated cjESCs, was obtained with efficiencies similar or even higher than that of ACTB. Although the KI efficiency among total transfected cjESCs were low, experimentally shown to be between 1.8–8.3% with Cas9-gRNA (Supplementary Figs S3b and S11b), the percentage of KI clones among cjESC clones that survived positive selection was over 80% in most cases (Figs 2e, 3f and Supplementary Fig. S7h), which is higher than that of previous studies recently reported using a similar strategy in human ESCs and induced pluripotent stem cells (iPSCs)^28–33^ and in macaque monkey ESCs (less than 50%)^34^. Additionally, in our method, we succeeded in obtaining over 10% of homozygous-KI clones in most cases (Figs 2e, 3f and Supplementary Fig. S7h). This was surprising, as homozygous KIs has been considered to be difficult to obtain using the conventional KI system in human iPSCs^33^.

We also observed an innate high HR activity of cjESCs, which may have also contributed to the extremely high KI efficiency revealed in this study. In the course of the current study, we observed a high KI ratio when targeting *PLP1* exon 1, even in the absence of site-directed DSB with Cas9-gRNA. We were able to obtain KI clones with 88.6% (31/35 clones) efficiency without negative selection, one of which was a homozygous KI clone (Fig. 2e and Supplementary Fig. S4). Since gene targeting efficiency of *PLP1* exon 1 in mouse ESCs was 2% using a similar construct^13^, we consider our result in cjESCs to be extraordinary. Although this high efficiency was not observed at other loci without Cas9-gRNA, an HR bias may have occurred in cjESCs. Further studies to explore the mechanism of HR in cjESCs will be useful for improving the KI efficiency in other species.

Since the *PLP1* gene is crucial for the stability of myelin which are formed by OLs^35^, its mutation, deletion, or duplication leads to a functional impairment of the central nervous system^14^. Some *PLP1* missense mutations cause severe phenotypes, yet null mutations or genetic deletions of the gene causes mild phenotypes^36^. Furthermore, the *PLP1* gene is thought to be associated with schizophrenia in mice and human^37,38^. Therefore, enabling genetic modification such as KO or introduction of missense mutation(s) in the *PLP1* gene in non-human primates, as performed in the current study, would help to generate new disease models for analysing motor functions and higher brain functions, which could be used for testing drug candidates and cell implantation in a preclinical setting.

*FOXP2* was originally identified as the gene responsible for hereditary language disorder^19^. As such, the gene is likely associated with the language acquisition of human beings during evolution, since two amino acid residues (N303 and S325) are specific to human *FOXP2*. Humanized *Foxp2* mice exhibit neuroanatomical changes of the striata and enhanced memory learning^39,40^. Since some groups suggested the existence of homologues of the Broca’s area in non-human primates, including marmosets^41–43^, *FOXP2* would be an interesting candidate for gene modification in the marmoset.

In addition, we also established KI technology in early-stage embryos of the common marmoset, which are based on both microinjection and electroporation methods. We succeeded in obtaining KI embryos with around 30% efficiency. In the future, this technology could be utilized for the generation of KI marmosets.

In conclusion, we developed an efficient KI method for marmoset ESCs and early-stage embryos. The method may be used to generate KI animals and for analyzing gene functions *in vitro* using a non-human primate model. In addition, the HR bias, which occurs after DSB in cjESCs and was observed in several experiments presented herein, should be studied thoroughly for future development of the KI technology. The findings in the current study would facilitate the use of non-human primate species (marmosets) as bridging models to fill the gap between mouse and human.

## Experimental Procedures

### Animals

All protocols for animal experiments were performed in accordance with the guidelines for laboratory animals set forth by the National Institutes of Health, and the Ministry of Education, Culture, Sports, Science and Technology (MEXT) of Japan, and were approved by the Institutional Animal Care and Use Committee of the RIKEN (approval No. H27-2-306(4)). Animal care was conducted in accordance with the National Research Council (NRC) Guide for the Care and Use of Laboratory Animals (2011). Marmosets used in the current study were 2–6-years-old (average weight from 250 to 450 g). The marmosets were pair/family-housed in a warm and humid condition (25 °C, 65%). In total, thirty female marmosets were used as oocyte donors and eleven male marmosets were used as sperm donors. The marmosets were obtained from the in-house breeding colony at RIKEN Institute. Oocyte donors were kept pairwise with vasoligated males. Oocyte and sperm collection, and *in vitro* fertilization (IVF) was performed as previously described^12^. In brief, for oocyte collection, the oocyte donors whose plasma progesterone levels were monitored were intramuscularly injected with recombinant human follicle-stimulating hormone (FSH, 25 IU; Fuji Pharma) for 9 days, followed by the intramuscular injection of human chorionic gonadotropin (hCG, 75 IU; ASKA Pharmaceutical) on day 10. On day 11 (16–20 hours after the hCG injection), the hormone-treated female marmosets were pre-anesthetized with 0.04 mg/kg medetomidine (Nippon Zenyaku), 0.40 mg/kg butorphanol (Meiji Seika Pharma) and 0.40 mg/kg midazolam (Astellas Pharma). The oocytes were surgically collected from the anesthetized animals. During the operation, the marmosets were inhalationally anesthetized with isoflurane (Sumitomo Dainippon Pharma). The collected oocytes were incubated in Porcine Oocyte Medium (Research Institute for the Functional Peptides) at 38 °C. For IVF, sperms were collected from healthy male marmosets. For insemination, each oocyte was incubated with 3.6 × 10^4^ sperms in a drop of TYH medium (LSI Medience) for 16 hours at 38 °C.

### Cell culture

Two common marmoset ES cell lines, No. 40 (female, 46XX) and DSY127 (male, 46XY) were used in the current study. The No. 40 line was established previously^44^, and DSY127 was kindly provided by Sumitomo Dainippon Pharma Co., Ltd. (Tokyo, Japan). These cells were cultured as described previously^45^. In brief, cjESCs were cultured on 30 Gy-irradiated mouse embryonic fibroblasts (MEFs) in ES medium (ESM) consisting of 1x KnockOut DMEM (Thermo Fisher) supplemented with 20% KnockOut Serum Replacement (Thermo Fisher), 1mM L-glutamine (Nakalai Tesque), 1% non-essential amino acids (Sigma), 0.2 mM 2-mercaptoethanol (Sigma) and 10 ng/ml fibroblast growth factor 2 (Peprotech). The KI cjESC lines generated in the current study will be distributed by the corresponding authors upon request.

### Transfection and genotyping

Double-stranded DNA targeting vectors (TVs) used for KI experiments in cjESCs were linearized by one-cut restriction enzyme before transfection. Each Cas9-gRNA vector and linearized TV was prepared at 1 μg/μl in Tris-HCl-EDTA buffer (pH 8.0). For transfection, a total of 10 μg of DNA was transfected, which consisted of the TV and Cas9-gRNA vector at a 4:1 molar ratio. For cjESC transfection, DNA vector(s) (total 10 μg), lipofectamine-LTX PLUS reagent (2.5 μl; Thermo Fisher) and LTX reagent (25 μl; Thermo Fisher) were diluted in 500 μl OPTI-MEM (Thermo Fisher), and added into sub-confluent cjESCs cultured in one well of a 6-well plate. 24 hours after transfection, the cells were dissociated and counted. 1 × 10^6^ cjESCs were suspended in ESM containing Y-27632 (10 μM; Merck Millipore), and re-seeded onto new feeder cells resistant to G418 or puromycin (day 1). On day 3, the medium was changed to ESM containing G418 (50 μg/ml; Thermo Fisher), or puromycin (1 μg/ml; Thermo Fisher) and Y-27632 (10 μM). Y-27632 was removed from the medium from day 5. After 2 weeks, the drug-resistant colonies were counted and picked for further cloning.

For genotyping by PCR and Southern blotting, the cloned ESCs were lysed overnight at 55 °C in cell lysis buffer consisting of Tris-HCl (0.2 M), EDTA (10 mM), SDS (0.2%) and NaCl (0.2 M) in nuclease-free water with proteinase K (10 μg/ml). Genomic DNA was purified using a standard method with phenol-chloroform and ethanol. PrimeSTAR Max DNA polymerase (Takara) was used for genotyping PCR, according to the manufacturer’s instructions. PCR was performed as follows: 30 s at 94 °C; 35 cycles of 10 s at 98 °C and 8 min at 68 °C; then 10 min at 68 °C; and a final incubation at 4 °C until gel electrophoresis. The primers are listed in Supplementary Table 1. Southern blotting was performed as described previously^45^. For digestion of genomic DNA, we used XbaI (*ACTB-EGFP*), BglII (*PLP1-EGFP*) and EcoRV (*FOXP2*, *PLP1*-P216S, S253T and A39T) (purchased from Takara or NEB). The entire images of the gels in Southern blotting analysis were appended in Supplementary Fig. S14.

### Transient selection

The procedure for the transfection of cjESCs is described above. After transfecting each TV or ssODN (8 μg) with the Cas9-gRNA vector (2 μg), the cjESCs were re-seeded onto new feeder cells (day 1). On day 2, the medium was changed to ESM. On day 3, the medium was changed to ESM containing puromycin (0.2 μg/ml) and Y-27632 (10 μM). On day 5, Y-27632 was removed from the medium. From day 6, the medium was changed to ESM every other day for further expansion.

### FACS analysis

FACS analysis was performed using FACSVerse flow cytometer (BD) with FACSuite software (BD) according to the manufacturer’s instructions. In brief, the cjESCs were expanded following transient selection, and the cells were suspended in FACS buffer consisting of fetal bovine serum (1%), EDTA (5 mM) and Y-27632 (10 μM) in 1x PBS buffer. 0.1% Propidium iodide staining solution (PI; Sigma) was used to remove dead cells.

### Genomic cleavage assay

Cas9-guided genomic cleavage assay was performed using GeneArt genomic cleavage detection kit (Thermo Fisher) according to the manufacturer’s instructions. Briefly, each Cas9-gRNA vector (10 μg) was introduced into cjESCs and then the cells were transiently selected. Genomic DNA was extracted from the cells for PCR. The resulting PCR mixtures were re-annealed under the following conditions: 5 min at 95 °C; 85 °C to 25 °C at −2 °C/s ramp speed; and 25 °C to 4 °C at −0.1 °C/s ramp speed. The re-annealed solutions were directly used for the cleavage reaction.

### Vector construction

#### Cas9-gRNA vectors

CRISPR-direct^46^ was used to design gRNA for marmoset genomic DNA. The gRNA sequences are listed in Supplementary Table 2. For the Cas9-gRNA vector, pSpCas9(BB)-2A-Puro (PX459) was used (a gift from Feng Zhang; Addgene plasmid # 48139).

#### TVs

The *ACTB-EGFP* vector was constructed previously^45^. The shortened *ACTB-EGFP* vectors were constructed based on the *ACTB-EGFP* vector. The genomic sequences of the putative marmoset *PLP1* and *FOXP2* genes were obtained from the *C. jacchus* genome database (Callithrix_jacchus-2.0.2: The Genome Sequencing Center at Washington University School of Medicine in St. Louis, http://genome.wustl.edu/pub/organism/Primate/Callithrix_jacchus/). To construct the *PLP1-EGFP* vector, a 3.2-kb fragment containing a region spanning upstream of the *PLP1* gene to the *PLP1* initiation codon, and a 5.0-kb fragment containing *PLP1* intron 1 were ligated with the vector pHNEO-EGFP, which harbours promoter-less *EGFP*, bovine growth hormone polyadenylation signal (pA), and floxed G418-resistance gene (Neo) under the mouse *Pgk-1* promoter (PGK).

To construct the *PLP1*-P216S and *PLP1-*S253T vectors, a 2.7-kb fragment containing *PLP1* introns 2 to 5, and a 4.2-kb fragment containing *PLP1* intron 5 and the downstream region of the *PLP1* gene, were ligated with pSINTK. The 2.7-kb fragment was mutagenized by PCR using specific primers to obtain the sequence encoding the Pro216Ser substitution (CCT > TCT), or the 4.2-kb fragment was mutagenized to obtain the sequence encoding the Ser253Thr substitution (TCC > ACC).

To construct the *PLP1*-A39T and *PLP1*-P15L vectors, a 2-kb fragment containing a region from *PLP1* intron 1 to intron 2, and a 1.4-kb fragment spanning *PLP1* intron 2 to intron 4, were subcloned into pDONR vectors, and introduced into pDEST-R3R4(R) with pENTR-L1-PGK-PuroTK-pA-L2^47^. The 2-kb fragment was mutagenized by PCR to generate A39T or P15L substitutions, and silent mutations to render the vectors to become undetectable by the gRNAs. For gene targeting experiments in the embryos, the *PGK-PuroTK* cassette was removed from the *PLP1*-P15L vector by Cre recombinase (NEB).

To construct the *FOXP2* TV, 2.6-kb and 1.3-kb fragments were subcloned into pDONR vectors, and introduced into pDEST-R3R4(R) with pENTR-L1-PGK-PuroTK-pA-L2^47^. The 2.6-kb fragment was mutagenized to encode human-specific variants (Thr301Asn and Asn323Ser).

All vectors were purified using plasmid DNA purification kit (Qiagen). The vectors used in the current study are listed in Supplementary Table 3. The listed vectors will be provided by Addgene (https://www.addgene.org) or the corresponding authors upon request.

### Neuronal induction

The cjESCs were induced into neuronal cells, including OLs, using a previously described method with slight modifications^48^. Briefly, dorsomorphin (3 μM; Sigma), SB431542 (3 μM; Tocris Bioscience) and CHIR99021 (3 μM; Wako) were added at days 1–3 of embryoid body formation. Immunochemical analysis was performed using Hoechst 33258 (Sigma) and the antibodies listed in Supplementary Table 4. The detailed experimental protocols will be provided by the corresponding authors upon request.

### Microinjection of the embryos

Marmoset embryos at the 2PN stage were prepared as described previously^12,49^. The microinjection solution consisted of annealed crRNA and tracrRNA (50 ng/μl; IDT), Cas9 protein (100 ng/μl; IDT) and TV (*PLP1*-P15L; 100 ng/μl), which were suspended in nuclease-free water. The TV was purified using QIAquick PCR purification kit (Qiagen). Approximately 5–10 pl of the injection solution was injected into the cytoplasm of the pronuclear stage embryos in M2 medium (Sigma). Following the microinjection, the embryos were cultured in ORIGIO sequential cleavage medium (Origio). The embryos that developed normally past the 8-cell stage were used for genotyping. The KAPA mouse genotyping kit (Kapa Biosystems) and PrimeSTAR Max DNA polymerase were used for embryo genotyping. Briefly, each developed embryo was washed in PBS drop once and then transferred into the extraction solution (3 μl) consisting of extraction buffer (1×) and extraction enzyme (2%), and was placed in the following conditions: 75 °C for 10 min, 95 °C for 5 min, and a final incubation at 4 °C. After extraction, PCR solution (22 μl) was added to the extract solution and centrifuged briefly before PCR reaction. The final PCR mixture (25 μl) contained the PrimeSTAR Max premix (1×) and primers (1.6 μM each). PCR was performed by temperature cycling as follows: 30 s at 94 °C; 40 cycle of 10 s at 98 °C and 150 s at 68 °C; 10 min at 68 °C; and a final incubation at 4 °C. A portion (4 μl) of the PCR solution was used for electrophoresis on 1% agarose gel, and the 1.5–2.2-kb DNA product band was extracted from the gel. The extracted PCR fragments were purified using a phenol-chloroform and ethanol method, and subcloned into pCR-BluntII-TOPO (Thermo Fisher) utilizing DH5αcompetent cells (Takara). Each cloned vector was sequenced using the BigDye Terminator v1.1 Cycle sequencing kit (Thermo Fisher) with the 3130xl DNA Analyzer (Applied Biosystems). For the RFLP analysis, a portion (4 μl) of the PCR mixture was used. The RFLP solution (20 μl) contained buffer L (1×), and *Apa*I or *Sac*I (1 μl; Takara). The RFLP reaction was performed as follows: 4 hours at 37 °C, followed by 20 min at 80 °C, and a final incubation at 4 °C until gel electrophoresis.

### Electroporation of the embryos

NEPA21 Super electroporator and CUY505P5 electrode (Nepa Gene) were used for electroporation. Marmoset embryos at the 2PN stage were transferred to an electroporation solution containing the Cas9 protein (100 ng/μl), annealed crRNA and tracrRNA (25 ng/μl in total), TV (100 ng/μl), and ssODN (100 ng/μl) in OPTI-MEM (1×; Thermo Fisher). The electroporation conditions were as follows. Poring pulse: 225 V, 2-pulse width, 50-ms pulse interval, four pulses, 10% attenuation rate, + . Transfer pulse: 20 V, 50-ms pulse width, 50-ms pulse interval, five pulses, 40% attenuation rate, +/−. The resistance value was adjusted to ca. 500 Ω just before electroporation. After the electroporation, the embryos were cultured in the ORIGIO sequential cleavage medium until development past the 8-cell stage.

### RT-PCR and qRT-PCR

RNA was isolated using the RNeasy mini kit (Qiagen) according to the manufacturer’s protocol. Total RNA (1.0 μg) was reverse-transcribed in the ReverTra Ace qPCR RT master mix (Toyobo). The resultant cDNAs were diluted in nuclease-free water (to 4 ng/μl). RT-PCR was performed using the PrimeSTAR Max DNA polymerase according to the manufacturer’s instructions. qRT-PCR was performed using the TB Green Premix Ex Taq II (Takara) on Viia 7 (Applied Biosystems) according to the manufacturer’s instructions. The primers used are listed in Supplementary Table 1.

### DNA sequencing

DNA sequencing analysis was performed using the BigDye Terminator v1.1 cycle sequencing kit (Thermo Fisher) with the 3130xl Genetic Analyzer (Applied Biosystems). The sequence data presented in the figures were illustrated using the Snap Gene software (GSL Biotech).

### Statistical analysis

All data are expressed as mean ± s.e.m. Differences between means were compared using the Student’s *t*-test. Differences were considered significant at *P* < 0.05.

## Supplementary information

Supplementary Information
