# Supplementary material for: Robust and efficient knock-in in embryonic stem cells and early-stage embryos of the common marmoset using the CRISPR-Cas9 system
Source: Sci Rep. 2019 Feb 6;9:1528. doi: 10.1038/s41598-018-37990-w (PMC6365532; doi:10.1038/s41598-018-37990-w)

Robust and efficient knock-in in embryonic stem cells and early-stage embryos of the common marmoset using the CRISPR-Cas9 system

Sho Yoshimatsu[1,2], Junko Okahara[3,4], Takefumi Sone[1], Yuta Takeda[3], Mari Nakamura[1], Erika Sasaki[4], Noriyuki Kishi[3], Seiji Shiozawa[1,3,*] & Hideyuki Okano[1,3,*]

[1]Department of Physiology, School of Medicine, Keio University, Shinjuku-ku, Tokyo 160-8582, Japan

[2]Laboratory for Proteolytic Neuroscience, RIKEN Center for Brain Science, Wako City, Saitama 351-0198, Japan

[3]Laboratory for Marmoset Neural Architecture, RIKEN Center for Brain Science, Wako City, Saitama 351-0198, Japan

[4]Central Institute for Experimental Animals, Kawasaki, Kanagawa 210-0821, Japan

Supplementary figure legends

Supplementary Figure S1. Genomic cleavage detection assay. (**a**) Schematic diagram of the marmoset *ACTB* gene sequence surrounding the 3′-UTR, and the corresponding gRNAs (ACTB-1, 2, 3). (**b**) Results of the GCA assay. All three gRNAs targeting *ACTB* 3′-UTR showed GCA. (**c**) Schematic diagram of the marmoset *PLP1* gene sequence surrounding exon 1, and the corresponding gRNAs (PLP1-1, 2, 3, 4). (**d**) The four gRNAs targeting the vicinity of *PLP1* exon 1 showed GCA. (**e**) Schematic diagram of the marmoset *PLP1* gene sequence surrounding intron 5, and the corresponding gRNAs (PLP1-ITR5-1, 2, 3). (**f**) The three gRNAs targeting *PLP1* intron 5 showed GCA. (**g**) Schematic diagram of the marmoset *PLP1* gene sequence in the vicinity of exon 2, and the corresponding gRNAs (PLP1-CDS2-1, 2, 3,4). (**h**) GCA analysis of gRNAs targeting the *PLP1* exon 2. Although the GCA of gRNA4 (PLP1-CDS2-4) seemed low, the other gRNAs showed obvious GCA. (**i**) Schematic diagram of the marmoset *FOXP2* gene sequence in the vicinity of exon 8, and the corresponding gRNAs (FOXP2-1, 2, 3, 4). (**j**) The four gRNAs targeting the vicinity of *FOXP2* exon 8 showed GCA.

Supplementary Figure S2 (related to Fig. 1e). Southern blotting analysis of the *ACTB-EGFP* ESCs. The EGFP++ and EGFP+ clones were analyzed by Southern blotting using the EGFP probe. Only one band corresponding to the ACTB-EGFP allele (8.9 kb) was detected. The separate images were cropped from the same gel. The entire image of the gel is shown in Supplementary Fig. S14b.

Supplementary Figure S3 (related to Fig. 1). FACS analysis of transiently-selected cjESCs (*ACTB-EGFP*). (**a-b**) Transiently-selected cjESCs, after further expansion, were analyzed by FACS. For transfection, the ACTB-EGFP TV (5'short and 3'short, shown in Fig. 1f, bottom) and the Cas9-gRNA vector harbouring the ACTB-2 gRNA were used for the gRNA(+) group, while the PX459 alone was used for the gRNA(–) group. The cjESCs were gated by FSC and SSC (P1, not shown) and the dead cells were removed by gating with PE-Cy7, which detects PI fluorescence (P2, not shown). The percentage of EGFP (GFP FITC)-positive cells was calculated for the P2-gated cjESCs. By detecting EGFP fluorescence using the P3 gate, EGFP(+) cells were 0.0% in WT cjESCs (not shown). This analysis was biologically and technically duplicated. (**c**) The percentage of EGFP-positive (EGFP+) cells in the gRNA(+) and gRNA(–) group. The percentage of EGFP-positive (EGFP+) cells in the gRNA+ group was 1.75 ± 0.17%, which was considered to be significant (P < 0.001) compared to that of the gRNA– group, 0.18 ± 0.05%.

Supplementary Figure S4 (related to Fig. 2c). Genotyping PCR of *PLP1* exon1. (**a**) 5′-region genotyping PCR of G418-resistant cjESC clones. The Cas9+ clone numbers are shown in grey (#1–39), and the Cas9– clone numbers are shown in black (#1–35). Ho, homozygous KI; He, heterozygous KI. (**b**) 3′-region genotyping PCR of G418-resistant clones. +, KI; –, non-KI.

Supplementary Figure S5 (related to Fig. 2). Validation of the *PLP1-EGFP* KI reporter. (**a**) Cre-mediated excision of the loxP-flanked *PGK-Neo-pA* cassette from the *PLP1*^{EGFP} allele. (**b**) Confirmation of the Cre-mediated cassette excision by genotyping PCR. (**c**) Schematic diagram of the differentiation procedure of cjESCs into neuronal cells, including oligodendrocytes (OLs). The upper numbers indicate days after differentiation. For the cjESCs, we used either the Cas9-#11 KI clone with its cassette excised or a WT clone. On day 0, the cjESCs were detached from MEFs and transferred to the floating culture. EB=embryoid body; 1st NS=primary neurosphere; 2nd NS=second neurosphere. (**d**) The *PLP-EGFP* cjESCs and the differentiated cells. Scale bars, 100, 50 and 50 μm (from left). (**e**) RT-PCR analysis of *PLP1*^{WT} or *PLP1*^{EGFP} cjESCs and differentiated cells from each cjESC (WT or EGFP) at day 70 (Diff). *GAPDH* was used as an internal control. (**f**) Immunofluorescence staining of mature OL markers (Galactocerebrosidase; GalC and Myelin basic protein; MBP) after a 70 days differentiation of *PLP1-EGFP* cjESCs. Scale bars, 100 μm (top), 50 μm (bottom).

Supplementary Figure S6 (related to Fig. 2). Genotyping PCR of G418-resistant cjESC colonies in bulk (*PLP1-EGFP*). (**a**) Genotyping PCR of G418-resistant cjESC colonies from each well following G418 selection of 1×10^6 cjESCs, which were transfected with the PLP1-EGFP TV, either with or without the Cas9-gRNA vector (gRNA: PLP1-2). The KI band intensity (PLP1^{EGFP} band intensity / PLP1^{WT} band intensity × 100) was quantified by the Image J software. (**b**) qRT-PCR analysis of G418-resistant cjESC colonies in bulk (*PLP1-EGFP*). RNA extraction was performed

simultaneously as genomic DNA extraction. Relative gene expression of *OCT4* and *NANOG* in WT cjESCs was set as 1.0. *GAPDH* was used as an endogenous control for quantification. (**c**) *PLP1* exon 1 targeting in male cjESCs. Genotyping PCR of the G418-resistant clones after G418 selection of male cjESCs (DSY127) transfected with the PLP1-EGFP TV. Hem; hemizygous KI.

Supplementary Figure S7. Introduction of missense mutations into the *PLP1* gene. (**a**) Schematic diagram of the *PLP1*-P216S and *PLP1*-S253T constructs. These TVs harboured 2.7-kb and 4.2-kb homology arms for the sequence surrounding intron 5 of the marmoset *PLP1* gene locus. P216S or S253T substitution mutations were introduced into exon 5 or exon 6 of the TVs. The loxP-flanked *PGK-Neo-pA* was placed between the homology arms. These TVs were not detected by gRNAs for intron 5 of the marmoset *PLP1* gene (PLP-ITR5-1, 2, 3). (**b**) The number of G418-resistant colonies following selection of 1×10^6 transfected cjESCs, shown as the mean ± s.e.m., n = 4. (**c**) 5′-region genotyping PCR of the G418-resistant clones using the 5′-external and 3′-internal primers. The separate images (gRNA3 #1-6, Control #1-2, WT) were cropped from the same gel. (**d**) 3′-region genotyping PCR of the G418-resistant cjESC clones using the 5′-primer for the selection cassette and the 3′-external primer. (**e**) Schematic diagram of the *PLP1*-A39T construct. The TV harbored 2.0-kb and 1.4-kb homology arms for the sequence surrounding intron 2 of the marmoset *PLP1* gene locus. The A39T mutation was introduced into exon 2 of the TV. The loxP-flanked *PGK-PuroTK-pA* was placed between the homology arms. These TVs were not detected

by gRNAs designed for exon 2 of the marmoset *PLP1* gene (PLP-CDS2-1, 2, 3, 4). (**f**) The number of puromycin-resistant colonies following selection of 1 × 10^6 transfected cjESCs, shown as the mean ± s.e.m., n = 3. (**g**) Genotyping PCR of the puromycin-resistant cjESC clones using the 5′- and 3′-external primer. The separate images (gRNA1 #1-5 and gRNA2 #1-6, gRNA2 #7-13) were cropped from each same gel respectively. (**h**) Summary of the gene targeting data for the *PLP1* exons 5, 6, and 2.

Supplementary Figure S8. DNA sequencing of the cjESCs with the *PLP1* missense mutation KI. (**a**) DNA sequencing analysis of the *PLP1*-P216S KI alleles of the KI cjESC clones gRNA1 #10 and gRNA2 #1. (**b**) DNA sequencing analysis of the *PLP1*-S253T KI alleles of the cjESC clones gRNA2 #1 and gRNA2 #2. (**c**) DNA sequencing analysis of the *PLP1*-A39T KI allele of the cjESC clone gRNA2 #3. The DNA sequencing results of other clones are summarized in Supplementary Table 5.

Supplementary Figure S9. Southern blotting analysis of the *PLP1*-P216S, -S253T, and -A39T KI cjESCs. Eleven KI cjESC clones and WT cjESCs were analyzed by Southern blotting. Genotypes of all analyzed clones matched the results of genotyping PCR. The separate images were cropped from the same gel. The entire image of the gel is shown in Supplementary Fig. S14c.

Supplementary Figure S10 (related to Fig. 3c). Genotyping PCR analysis of the puromycin-resistant cjESC clones (*FOXP2* humanization). All clones transfected with

gRNA1-3, and clones #10-15 that were transfected with gRNA4 were shown in Fig. 3c. The separate images (gRNA4 #1-3 and #4-9) were cropped from the same gel.

Supplementary Figure S11 (related to Fig. 4). RFLP analysis of *PLP1*-P15L KI in cjESCs. (**a**) The *PLP1*-P15L TV or ssODN, and Cas9-gRNA vector (gRNA: PLP1-CDS2-2) were transfected into cjESCs followed by transient selection. Following expansion, the genomic DNA was extracted and analyzed by RFLP. PCR was performed using the 5′-external and 3′-internal primers, which were also used for genotyping PCR analysis for early-stages embryos shown in Fig. 4c, S12b and S13b (Supplementary Table 1). The PCR fragment was digested by *Apa*I (WT allele) or *Sac*I (KI allele). (**b**) RFLP analysis of the PLP1 exon 2 using the genomic DNA extracted from WT cjESCs and the transfected cjESCs, which underwent transient selection. White arrows indicate the KI allele detected by *Sac*I digestion of the PCR fragment. The KI efficiency was calculated according to the band intensity and DNA fragment size (intact band: 2126 bp, SacI-digested band: 1560 bp). KI efficiency (%) = (SacI-digested band intensity / total band intensity) $\times$ 1.362 × 100. The band intensity was quantified by the Image J software. This experiment was biologically duplicated (N1 and N2).

Supplementary Figure S12 (related to Fig. 4). Analysis of the microinjected embryos. (**a**) Summary of *PLP1* gene targeting by microinjection of the early-stage marmoset embryos. (**b**) RFLP analysis of the microinjected embryos at the 8-cell stage or beyond. White arrows indicate the KI allele detected by *Sac*I digestion of the PCR fragments. (**c**)

DNA sequencing analysis of subcloned PCR fragments. The number of subclones that carried the sequence shown in the right side of each lane among the total number of subclones analyzed is shown as a fractional number (number of subclones with the sequence shown/ total subclone number). Bases in red letters indicate that the sequence is different from the WT allele. At least seven subclones were analyzed for each embryo.

Supplementary Figure S13. Analysis of the electroporated embryos. (**a**) Summary of *PLP1* gene targeting by electroporation of early-stage marmoset early embryos. (**b**) RFLP analysis of the electroporated embryos at the 8-cell stage or beyond. The separate images of the uncut samples (top) were cropped from different gels. White arrows indicate the KI allele detected by *Sac*I digestion of the PCR fragments. The separated images of the *Sac*I/*Apa*I cut samples were cropped from the same gel. (**c**) Subcloned DNA sequencing analysis of the electroporated embryos at the 8-cell stage or beyond.

Supplementary Figure S14. Entire images of gels in Southern blotting analysis. (**a**) The entire image of the gel shown in Fig. 1e. Unrelated samples were loaded in the two lanes between the EGFP++ clone and DNA marker. (**b**) The entire image of the gel shown in Supplementary Fig. S2. Unrelated samples were loaded in the two lanes between the EGFP++ clone and DNA marker. (**c**) The entire image of the gel shown in Supplementary Fig. S9. An unrelated sample was loaded in lane 12 (the right lane of the lane 11, the lane number is shown in the Supplementary Fig.S9).

Supplementary tables

Supplementary Table 1: Primers.

Gene	Usage	Primer sequence
ACTB	Cleavage detetion	AAAACTGGAACGGTGAAGGTGAC
		TCTAAGGCTGCTCAATGTCAAGG
PLP1	Cleavage detection	GGGAGCCCTTAGAAAAGGGAGTA
		TTGGAACCAAGGGAAAAGAGAAG
PLP1	Cleavage detection	ATCTCACATGCTGAAAGCCAAAA
		TTAGTGCCTCTCTTGGGTTCTCC
PLP1	Cleavage detection	ATCCATCTGCAAAACAGCTGAGG
		GTCAACTCACCAGGGAAACCAGA
FOXP2	Cleavage detection	TTTGGTGCTGGTGGTGTAAAAGT
		GGCAAACCAGATTTTCCATCCTA
ACTB	Genotyping PCR	CGTTCCGAAAGTTGCCTTTTATG
		CTTGGCCTCATTTTTAAGGTGTG
PLP1	Genotyping PCR, Exon1, 5' region	CCAGGGCTAGGAAAGCTGTAAGA
		AGAAAACAAGTGCCAAATCACGA
PLP1	Genotyping PCR, Exon1, 3' region	TAGTTGCCAGCCATCTGTTGTTT
		GGCTAAGTGGTTGTGCAGGTATG
PLP1	Genotyping PCR, Exon2	TTCGGTCTTTCCAAATCTGTTCA
		AGGAGGATCCCTGGGTTAGAAAG
PLP1	Genotyping PCR, Exon2 (Embryo) and RFLP analysis	GCCATTCTTTTCCCCATCTAGTCA
		CAGGAGTGGGGAGCTAGGTAAGA
PLP1	Genotyping PCR, Exon5,6, 5' region	GGTCACAGGGTAAGCAAGATGTG
		CCCATAACACCCAGAACACAGAA
PLP1	Genotyping PCR, Exon5,6, 3' region	GGTCACAGGGTAAGCAAGATGTG
		CCCATAACACCCAGAACACAGAA
FOXP2	Genotyping PCR	ACCTGCCTCACCTGACACACTTA
		ACGAGCTGTAGCAAAAGAACACG
ACTB	Southern blotting	CATTCAGCAGCGTGGAGCTC

		AACTTTCCCAGCCTGTCTAC
PLP1	Southern blotting, Exon1	GGCAGACCCTTCAGAGACAG
		GGGAATTAGGGGTGTGGAGT
PLP1	Southern blotting, Exon2,5,6	AACAGAATCAGGCAAAGGAGTTC
		AGCAAATGCCAAGTAGATGACTG
FOXP2	Southern blotting	GCAAGAGTTGGCAATAGAGCAGA
		TGGCAATTACCCAATAGCAACAG
EGFP	Southern blotting	ATGGTGAGCAAGGGCGAGGA
		TTACTTGTACAGCTCGTCCATG
PLP1	DNA Sequencing, Exon2	ATCTCACATGCTGAAAGCCAAAA
PLP1	DNA Sequencing, Exon5	CCTACCCACTATGGAAGCAC
PLP1	DNA Sequencing, Exon6	CCTCCTCTTTCCTCATACCACTT
FOXP2	DNA Sequencing	TTAAATGGAACATCAAGTGA
GAPDH	RT-PCR and qRT-PCR	GCACCGTCAAGGCTGAGAAC
		TGGTGAAGACGCCAGTGGA
PLP1	RT-PCR	TCAATGTGATCCATGCCTTCCAG
		CTGGCCCCCTGTTACCGTTG
OCT4	qRT-PCR	GGAGGAAGCTGACAACAATGAAA
		GGCCTGCACGAGGGTTT
NANOG	qRT-PCR	ACGAACATGCCACCTGAAGA
		TACGAGGAAGGGGAGGAGGT
EGFP	RT-PCR	ATGGTGAGCAAGGGCGAGGA
		TTACTTGTACAGCTCGTCCATG

Supplementary Table 2: gRNA sequence (including PAM sequence).

Gene	Cleavage Region	gRNA Name	gRNA sequence
ACTB	3'UTR	ACTB-1	AGTTCTCTCCCAAGTCCACACGG

ACTB	3'UTR	ACTB-2	AAAAGCCTTCATACATCTCGAGG
ACTB	3'UTR	ACTB-3	AAGCCTTCATACATCTCGAGGG
PLP1	5'UTR	PLP1-1	TGAAACTTACCCATGTCTTTGG
PLP1	Intron1	PLP1-2	GAATTTCCAACTTTGGGGTTCGG
PLP1	Intron1	PLP1-3	TTGGGGTTCGGGGGTTCCAATGG
PLP1	Intron1	PLP1-4	GGTTTACATGAGTCGTGATTTGG
PLP1	Exon2	PLP1-CDS2-1	CTGTGCAAGATGTCTGGTAGGGG
PLP1	Exon2	PLP1-CDS2-2	GCTGTGCAAGATGTCTGGTAGGG
PLP1	Exon2	PLP1-CDS2-3	TGATGAGATACTCATAGTCCTGG
PLP1	Exon2	PLP1-CDS2-4	CTATTTCTCCAAAAACTACCAGG
PLP1	Intron5	PLP1-ITR5-1	GGCCCCTAGGCACGACTGTAGGG
PLP1	Intron5	PLP1-ITR5-2	GGTTCCCTACAGTCGTGCCTAGG
PLP1	Intron5	PLP1-ITR5-3	GTTCCCTACAGTCGTGCCTAGGG
FOXP2	Intron8	FOXP2-1	TTCAAATACGAAAAGTTGGTTGG
FOXP2	Intron8	FOXP2-2	AAAAGTTGGTTGGATATAGTTGG
FOXP2	Intron8	FOXP2-3	GCAGTTCAAATACGAAAAGTTGG
FOXP2	Exon8	FOXP2-4	TGGTGGTGATGCTTTGGAAGTGG

Supplementary Table 3: Vectors.

Usage	Vector Name	Size
ACTB targeting	pKI-cjACTB-EGFP	13646bp
ACTB targeting	pKI-cjACTB-EGFP-5'short	12731bp
ACTB targeting	pKI-cjACTB-EGFP-3'short	10023bp
ACTB targeting	pKI-cjACTB-EGFP-5'3'short	9108bp

PLP1 targeting (Exon1)	pKI-cjPLP1e1-EGFP	16456bp
PLP1 targeting (Exon2)	pKI-cjPLP1e2-A39T	8819bp
PLP1 targeting (Exon2)	pKI-cjPLP1e2-P15L	8819bp
PLP1 targeting (Exon5)	pKI-cjPLP1e5-P216S	14132bp
PLP1 targeting (Exon6)	pKI-cjPLP1e5-S253T	14132bp
PLP1 targeting (Embryo)	pKI-cjPLP1e2-P15L-Δlox	6243bp
FOXP2 targeting	pKI-cjFOXP2-hum	9358bp

Supplementary Table 4: Antibodies.

Name	Target	Manufacturer	Clone	Host-Type	Dillution
Anti-MBP antibody	MBP	BioRad	MCA409S	Rat IgG2a	1:1000
Anti-Galactocerebroside antibody	GalC	Millipore	MAB342	Ms IgG3	1:1000
Goat anti-Ms IgG Alexa Fluor 555	Ms IgG	Thermo Fischer	A-21424	Goat	1:1000
Goat anti-Rat IgG Alexa Flour 647	Rat IgG	Thermo Fischer	A-21247	Goat	1:1000

Supplementary Table 5: Summary of DNA sequencing analysis of mutation KI ESCs.

Gene	Mutation	gRNA	Clone#	Genotype (PCR)	Sequence variation (in KI allele)
PLP1	P216S	PLP1-ITR5-1	#1	Homo-KI	P216S/WT
PLP1	P216S	PLP1-ITR5-1	#2	Hetero-KI	P216S
PLP1	P216S	PLP1-ITR5-1	#3	Hetero-KI	WT
PLP1	P216S	PLP1-ITR5-1	#4	Hetero-KI	P216S/WT (mosaic)
PLP1	P216S	PLP1-ITR5-1	#5	Hetero-KI	P216S/WT (mosaic)
PLP1	P216S	PLP1-ITR5-1	#6	Hetero-KI	P216S/WT (mosaic)
PLP1	P216S	PLP1-ITR5-1	#7	Hetero-KI	P216S
PLP1	P216S	PLP1-ITR5-1	#8	Hetero-KI	WT
PLP1	P216S	PLP1-ITR5-1	#9	Hetero-KI	WT
PLP1	P216S	PLP1-ITR5-1	#10	Homo-KI	P216S/WT

PLP1	P216S	PLP1-ITR5-1	#11	Hetero-KI	P216S
PLP1	P216S	PLP1-ITR5-1	#12	Hetero-KI	P216S
PLP1	P216S	PLP1-ITR5-2	#1	Homo-KI	P216S
PLP1	P216S	PLP1-ITR5-2	#2	Hetero-KI	P216S/WT (mosaic)
PLP1	P216S	PLP1-ITR5-2	#3	Homo-KI	P216S/WT
PLP1	P216S	PLP1-ITR5-2	#4	Homo-KI	P216S/WT
PLP1	P216S	PLP1-ITR5-2	#5	Homo-KI	WT/WT
PLP1	P216S	PLP1-ITR5-2	#6	Homo-KI	WT
PLP1	P216S	PLP1-ITR5-2	#7	Homo-KI	P216S
PLP1	P216S	PLP1-ITR5-2	#8	Homo-KI	P216S
PLP1	P216S	PLP1-ITR5-2	#9	Hetero-KI	P216S
PLP1	P216S	PLP1-ITR5-2	#10	Hetero-KI	WT
PLP1	P216S	PLP1-ITR5-2	#11	Hetero-KI	P216S
PLP1	P216S	PLP1-ITR5-2	#12	Homo-KI	P216S/WT
PLP1	P216S	PLP1-ITR5-3	#1	Hetero-KI	P216S
PLP1	P216S	PLP1-ITR5-3	#2	Homo-KI	P216S/WT
PLP1	P216S	PLP1-ITR5-3	#3	Hetero-KI	P216S
PLP1	P216S	PLP1-ITR5-3	#4	Hetero-KI	P216S
PLP1	P216S	PLP1-ITR5-3	#5	Hetero-KI	P216S
PLP1	P216S	PLP1-ITR5-3	#6	Hetero-KI	P216S/WT (mosaic)
PLP1	P216S	PLP1-ITR5-3	#7	Homo-KI	P216S/WT
PLP1	P216S	PLP1-ITR5-3	#8	Homo-KI	WT
PLP1	P216S	PLP1-ITR5-3	#9	Homo-KI	P216S
PLP1	P216S	PLP1-ITR5-3	#10	Hetero-KI	P216S
PLP1	P216S	PLP1-ITR5-3	#11	Hetero-KI	P216S
PLP1	P216S	PLP1-ITR5-3	#12	Hetero-KI	P216S
PLP1	S253T	PLP1-ITR5-2	#1	Homo-KI	S253T
PLP1	S253T	PLP1-ITR5-2	#2	Hetero-KI	S253T
PLP1	S253T	PLP1-ITR5-2	#3	Homo-KI	S253T
PLP1	S253T	PLP1-ITR5-2	#4	Hetero-KI	S253T
PLP1	S253T	PLP1-ITR5-2	#5	Hetero-KI	WT
PLP1	S253T	PLP1-ITR5-2	#6	Hetero-KI	S253T

PLP1	A39T	PLP1-CDS2-1	#1	Hetero-KI	A39T
PLP1	A39T	PLP1-CDS2-1	#2	Hetero-KI	A39T
PLP1	A39T	PLP1-CDS2-1	#4	Homo-KI	A39T/WT
PLP1	A39T	PLP1-CDS2-1	#5	Hetero-KI	A39T/WT (mosaic)
PLP1	A39T	PLP1-CDS2-2	#2	Hetero-KI	A39T
PLP1	A39T	PLP1-CDS2-2	#3	Homo-KI	A39T
PLP1	A39T	PLP1-CDS2-2	#4	Homo-KI	A39T
PLP1	A39T	PLP1-CDS2-2	#5	Homo-KI	A39T
PLP1	A39T	PLP1-CDS2-2	#6	Hetero-KI	A39T
PLP1	A39T	PLP1-CDS2-2	#7	Homo-KI	A39T
PLP1	A39T	PLP1-CDS2-2	#8	Hetero-KI	A39T
PLP1	A39T	PLP1-CDS2-2	#9	Homo-KI	A39T
PLP1	A39T	PLP1-CDS2-2	#10	Homo-KI	A39T
PLP1	A39T	PLP1-CDS2-2	#11	Homo-KI	A39T
PLP1	A39T	PLP1-CDS2-2	#12	Homo-KI	A39T
PLP1	A39T	PLP1-CDS2-2	#13	Hetero-KI	A39T
PLP1	A39T	PLP1-CDS2-3	#1	Homo-KI	A39T
PLP1	A39T	PLP1-CDS2-3	#4	Homo-KI	A39T
PLP1	A39T	PLP1-CDS2-3	#5	Hetero-KI	A39T
PLP1	A39T	PLP1-CDS2-3	#6	Homo-KI	A39T
PLP1	A39T	PLP1-CDS2-4	#1	Hetero-KI	A39T
PLP1	A39T	PLP1-CDS2-4	#2	Homo-KI	A39T
PLP1	A39T	PLP1-CDS2-4	#3	Hetero-KI	A39T
PLP1	A39T	PLP1-CDS2-4	#4	Hetero-KI	A39T/WT (mosaic)
PLP1	A39T	PLP1-CDS2-4	#5	Hetero-KI	A39T/WT (mosaic)
PLP1	A39T	PLP1-CDS2-4	#6	Hetero-KI	A39T
FOXP2	Hum	FOXP2-1	#1	Hetero-KI	WT
FOXP2	Hum	FOXP2-1	#2	Hetero-KI	WT
FOXP2	Hum	FOXP2-1	#3	Hetero-KI	WT
FOXP2	Hum	FOXP2-1	#4	Hetero-KI	T301N/WT + N323S/WT (mosaic)
FOXP2	Hum	FOXP2-1	#5	Hetero-KI	WT

FOXP2	Hum	FOXP2-1	#6	Hetero-KI	WT
FOXP2	Hum	FOXP2-2	#1	Hetero-KI	WT
FOXP2	Hum	FOXP2-2	#2	Hetero-KI	T301N/WT + N323S/WT (mosaic)
FOXP2	Hum	FOXP2-2	#3	Hetero-KI	T301N/WT + N323S/WT (mosaic)
FOXP2	Hum	FOXP2-2	#4	Hetero-KI	T301N/WT + N323S/WT (mosaic)
FOXP2	Hum	FOXP2-2	#5	Hetero-KI	T301N/WT + N323S/WT (mosaic)
FOXP2	Hum	FOXP2-2	#6	Hetero-KI	T301N/WT + N323S/WT (mosaic)
FOXP2	Hum	FOXP2-3	#1	Hetero-KI	T301N/WT + N323S/WT (mosaic)
FOXP2	Hum	FOXP2-3	#2	Hetero-KI	T301N/WT + N323S/WT (mosaic)
FOXP2	Hum	FOXP2-3	#3	Hetero-KI	T301N/WT + N323S/WT (mosaic)
FOXP2	Hum	FOXP2-3	#4	Hetero-KI	T301N/WT + N323S/WT (mosaic)
FOXP2	Hum	FOXP2-3	#5	Hetero-KI	T301N/WT + N323S/WT (mosaic)
FOXP2	Hum	FOXP2-3	#6	Hetero-KI	T301N/WT + N323S/WT (mosaic)
FOXP2	Hum	FOXP2-4	#2	Homo-KI	T301N + N323S
FOXP2	Hum	FOXP2-4	#3	Hetero-KI	T301N + N323S
FOXP2	Hum	FOXP2-4	#4	Homo-KI	T301N + N323S
FOXP2	Hum	FOXP2-4	#5	Homo-KI	T301N + N323S
FOXP2	Hum	FOXP2-4	#6	Hetero-KI	T301N + N323S
FOXP2	Hum	FOXP2-4	#7	Homo-KI	T301N + N323S
FOXP2	Hum	FOXP2-4	#8	Homo-KI	T301N + N323S
FOXP2	Hum	FOXP2-4	#9	Hetero-KI	T301N + N323S

FOXP2	Hum	FOXP2-4	#11	Hetero-KI	T301N + N323S
FOXP2	Hum	FOXP2-4	#12	Homo-KI	T301N + N323S
FOXP2	Hum	FOXP2-4	#13	Hetero-KI	T301N + N323S
FOXP2	Hum	FOXP2-4	#14	Hetero-KI	T301N + N323S
FOXP2	Hum	FOXP2-4	#15	Homo-KI	T301N + N323S
FOXP2	Hum	FOXP2-4	#16	Hetero-KI	T301N + N323S
FOXP2	Hum	FOXP2-4	#17	Hetero-KI	T301N + N323S
FOXP2	Hum	FOXP2-4	#18	Hetero-KI	T301N + N323S
FOXP2	Hum	FOXP2-4	#19	Homo-KI	T301N + N323S
FOXP2	Hum	FOXP2-4	#20	Hetero-KI	T301N + N323S
FOXP2	Hum	FOXP2-4	#21	Hetero-KI	T301N + N323S
FOXP2	Hum	FOXP2-4	#22	Hetero-KI	T301N + N323S
FOXP2	Hum	FOXP2-4	#23	Hetero-KI	T301N + N323S
FOXP2	Hum	FOXP2-4	#24	Hetero-KI	T301N + N323S
FOXP2	Hum	FOXP2-4	#26	Hetero-KI	T301N + N323S
FOXP2	Hum	FOXP2-4	#27	Hetero-KI	T301N + N323S
FOXP2	Hum	FOXP2-4	#28	Homo-KI	T301N + N323S
FOXP2	Hum	FOXP2-4	#29	Hetero-KI	T301N + N323S
FOXP2	Hum	FOXP2-4	#30	Hetero-KI	T301N + N323S
FOXP2	Hum	FOXP2-4	#32	Hetero-KI	T301N + N323S

Supplementary sequence

PLP1 P15L ssODN (200bp)

Modified sequences from the WT sequence was shown in lower case.

GCTTTGAGTGGCATGAGCTACCTACTGGATGTGCCTGACTGTTTCCCCTTCTTCTT
CCCCAGGCTTGTTAGAATGCTGTGCAAGATGTCTGGTtGGaGCtCtCTTcGCTTCCCT
GGTGGCCACTGGATTGTGTTTCTTTGGGGTGGCACTGTTCTGTGGCTGTGGACAT
GAAGCCCTCACTGGCACAGAAAAGCTAATTG

Supplementary Figure S1

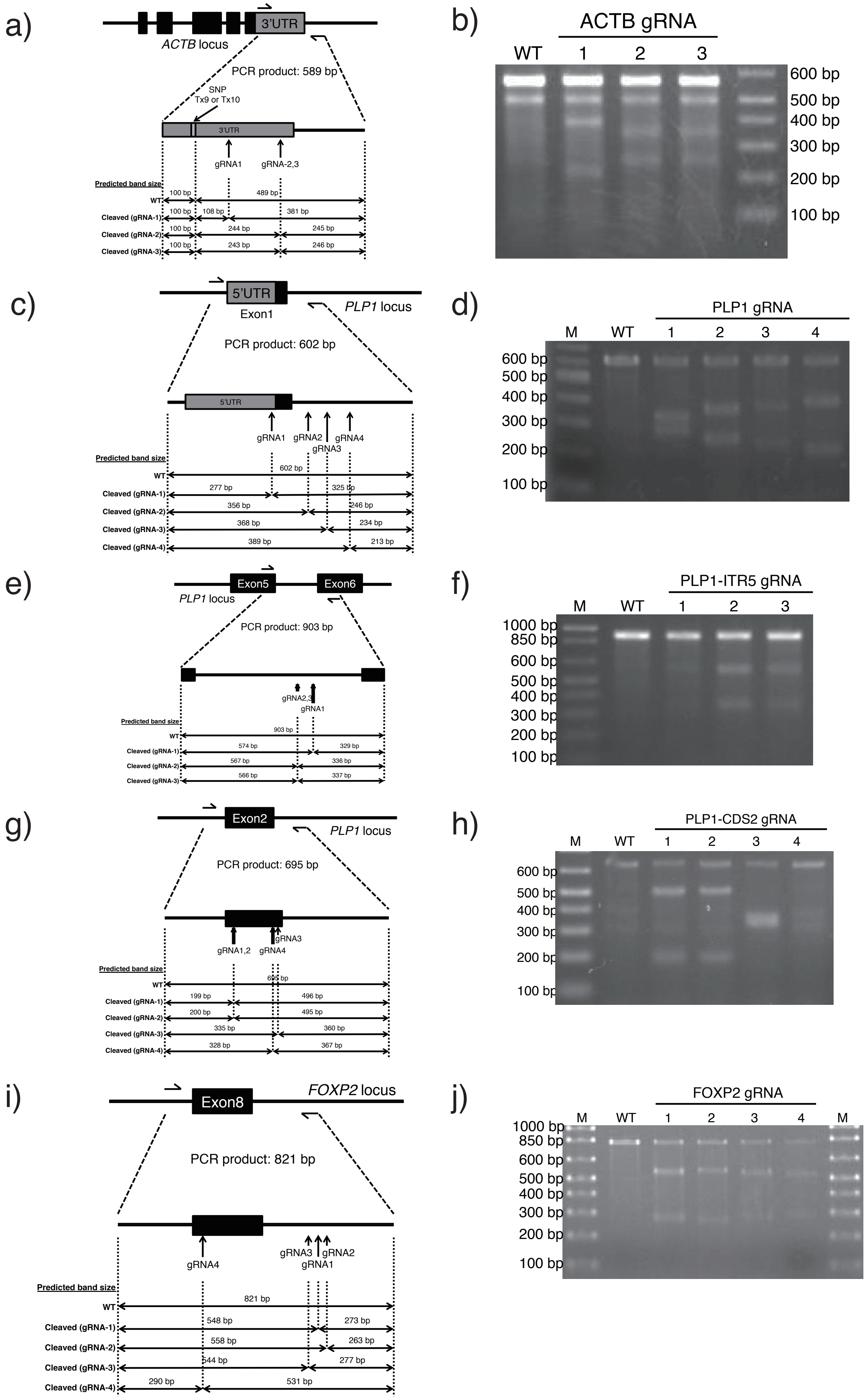

Supplementary Figure S2

Southern analysis of G418 resistant cjESC clones (ACTB-EGFP)
probe: EGFP probe

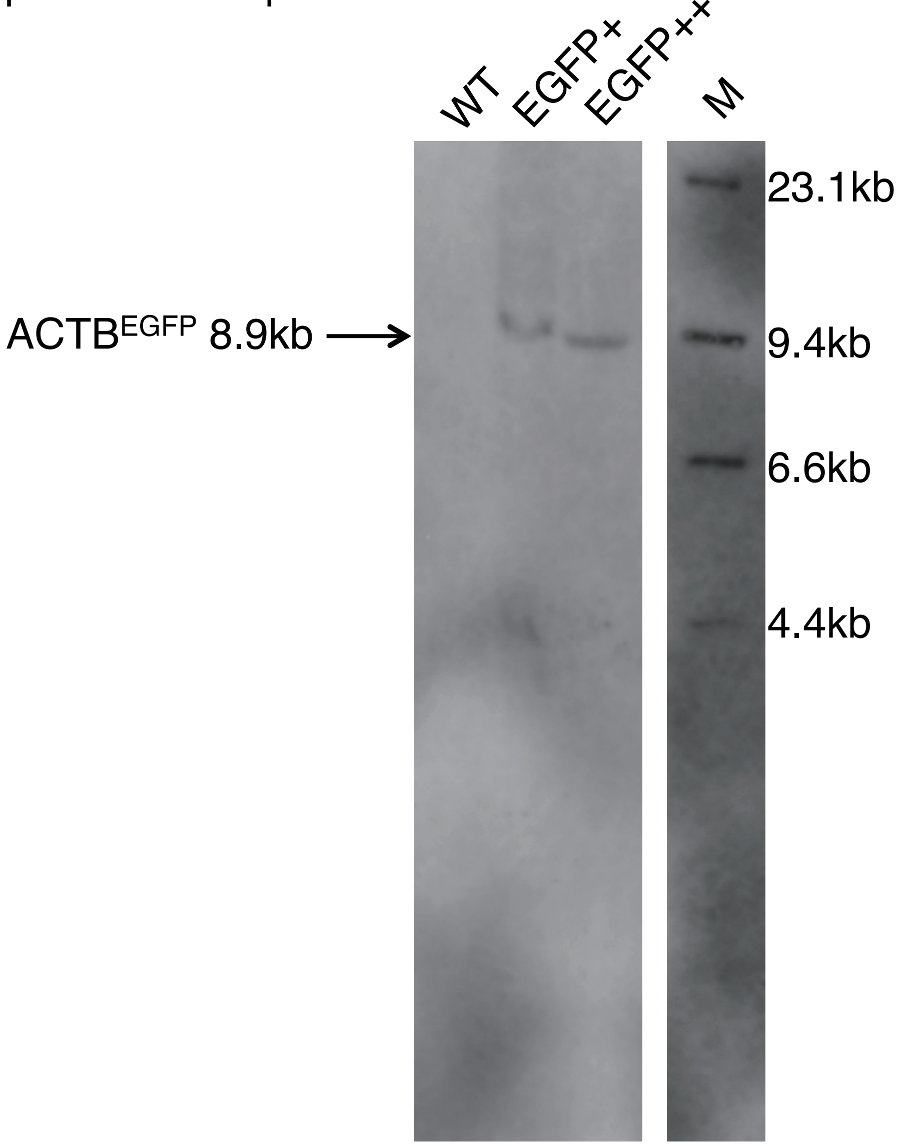

Supplementary Figure S3

a) ACTB-EGFP gRNA(-)
EGFP(+): 0.18 ± 0.05%

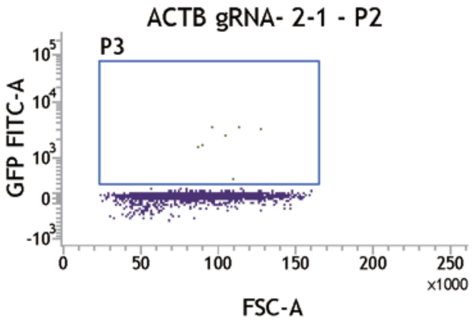

b) ACTB-EGFP gRNA(+)
EGFP(+): 1.75 ± 0.17%

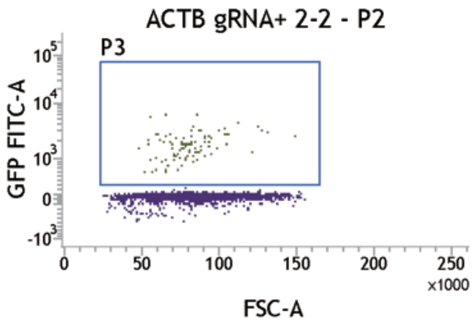

c)

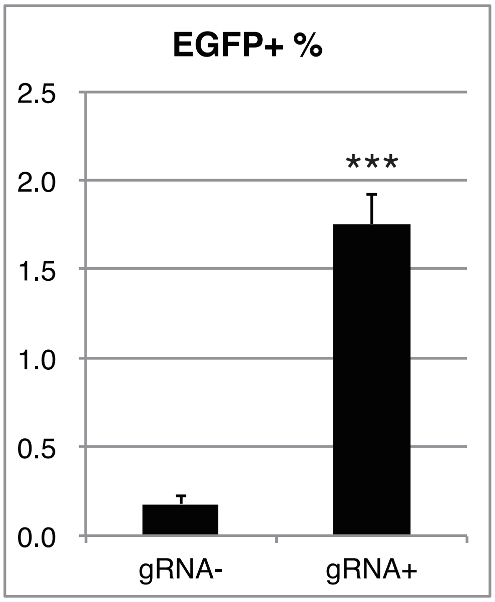

Supplementary Figure S4

a)

Genotyping PCR analysis of G418 resistant cjESC clones (PLP1-EGFP, **5' side**)
Cas9+ clone (gray #)
Cas9- clone (black #)

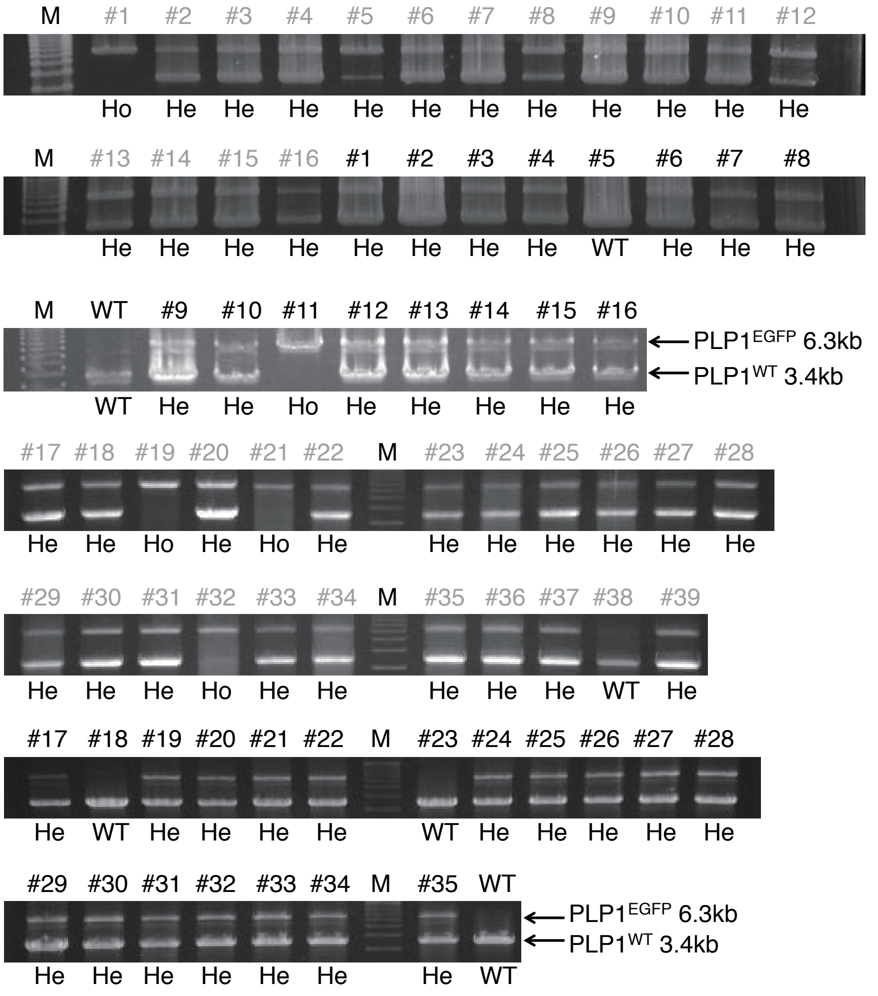

b)

Genotyping PCR analysis of G418 resistant cjESC clones (PLP1-EGFP, **3' side**)
Cas9+ clone #
Cas9- clone #

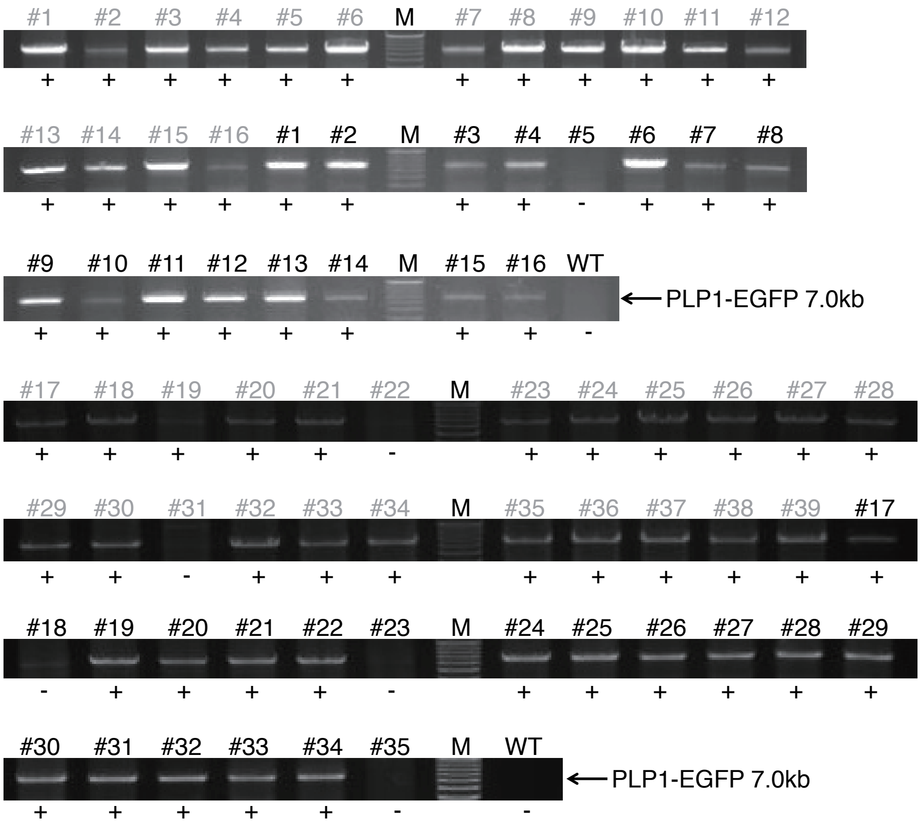

Supplementary Figure S5

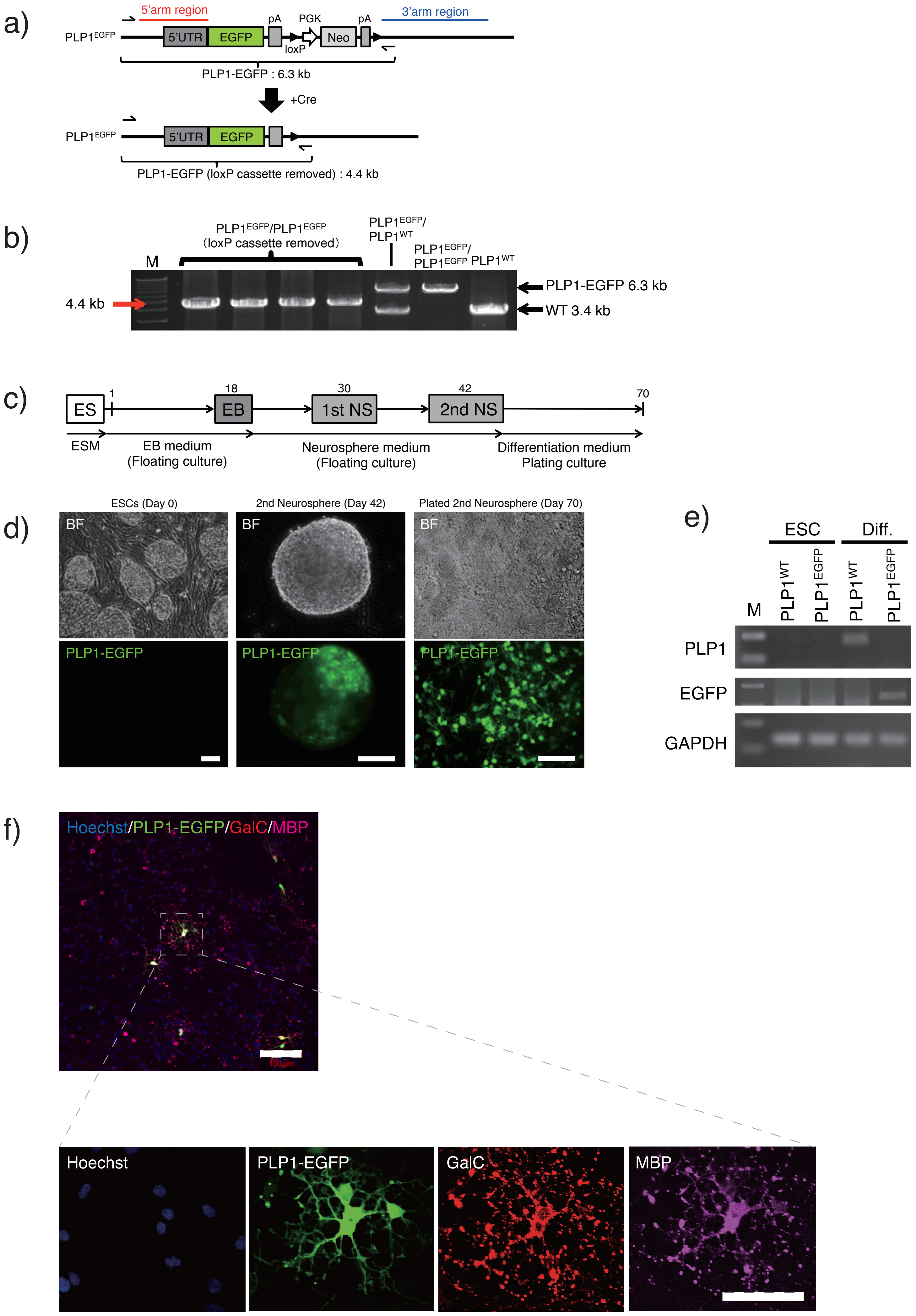

Supplementary Figure S6

a)

Genotyping PCR analysis of G418 resistant cjESC bulk colonies (PLP1-EGFP TV trasfected)

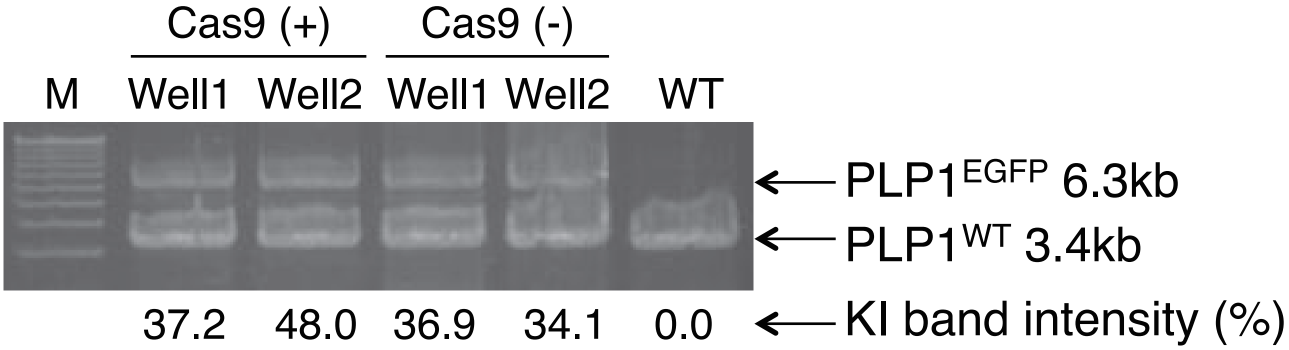

b)

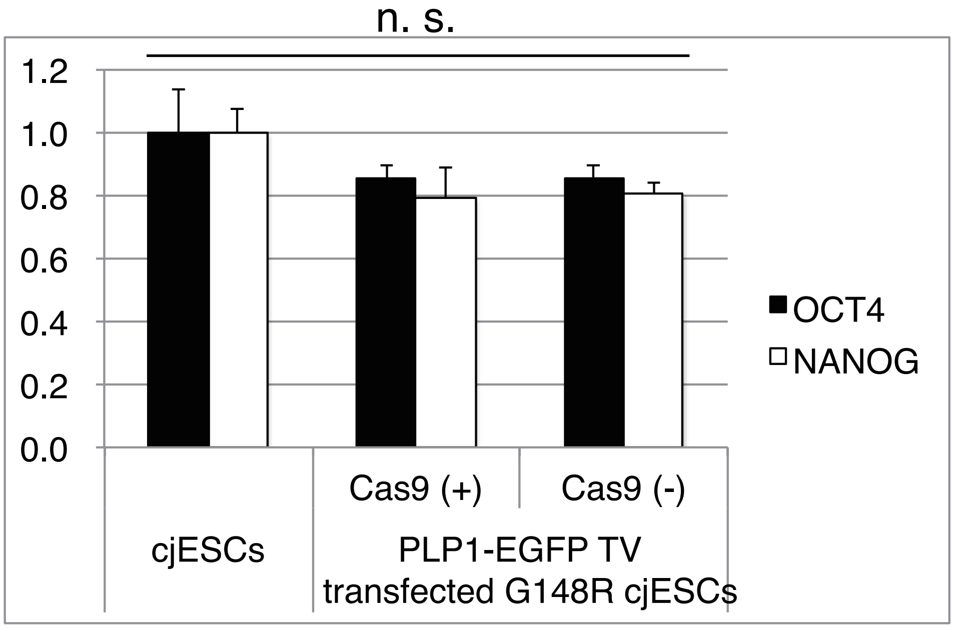

c)

Genotyping PCR analysis of DSY127 cjESC G418 resisntant clones (PLP1-EGFP TV transfected, without Cas9)

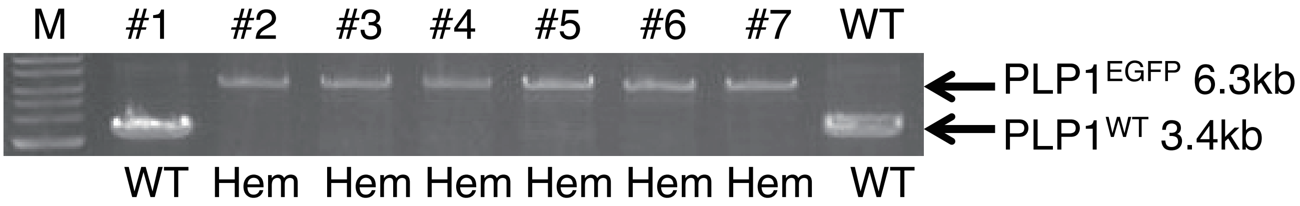

Supplementary Figure S7

a) b) c) d)

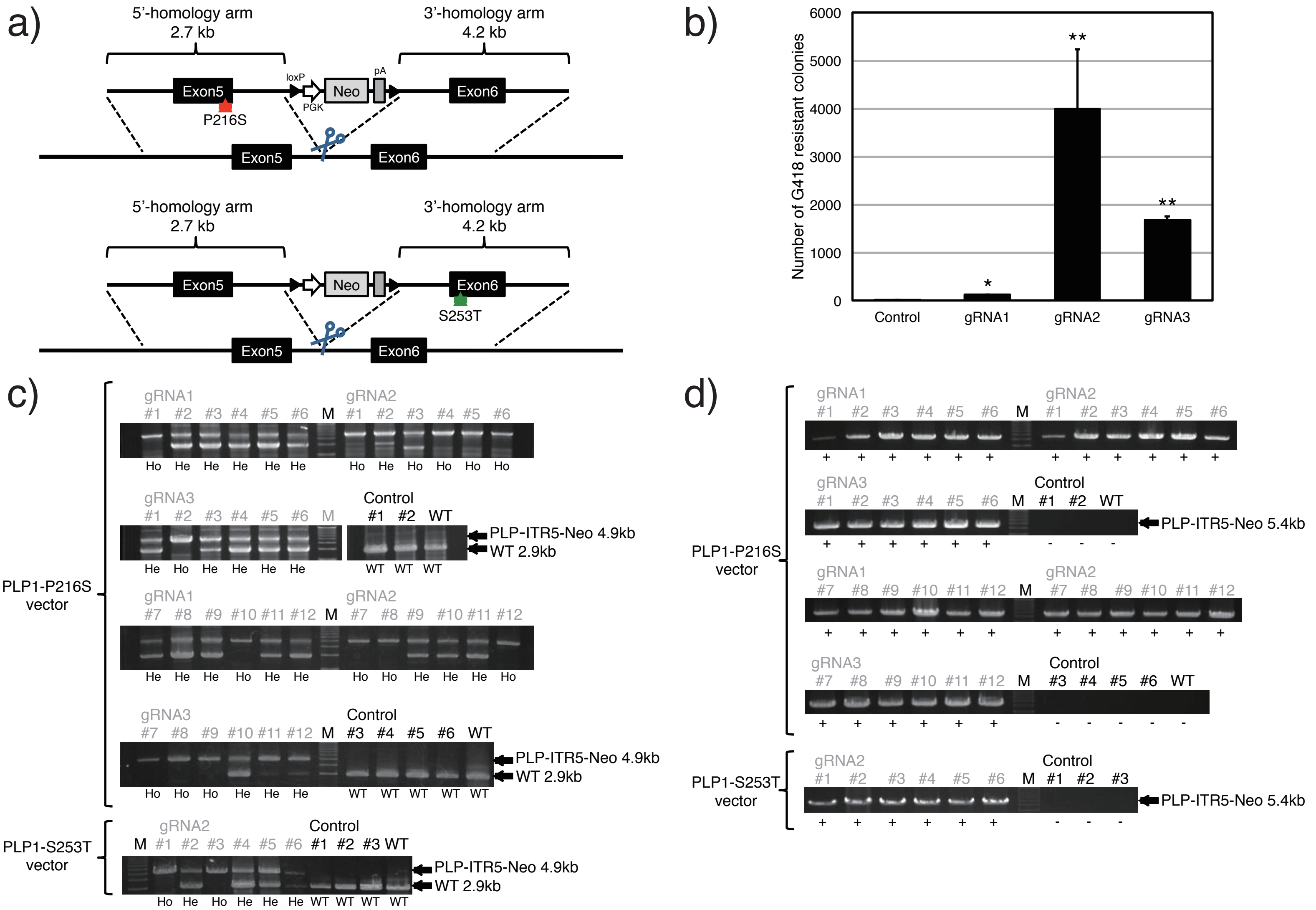

e)

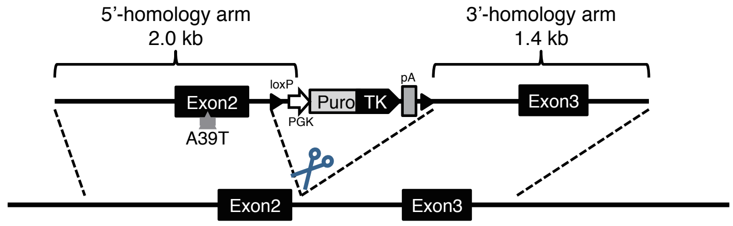

f)

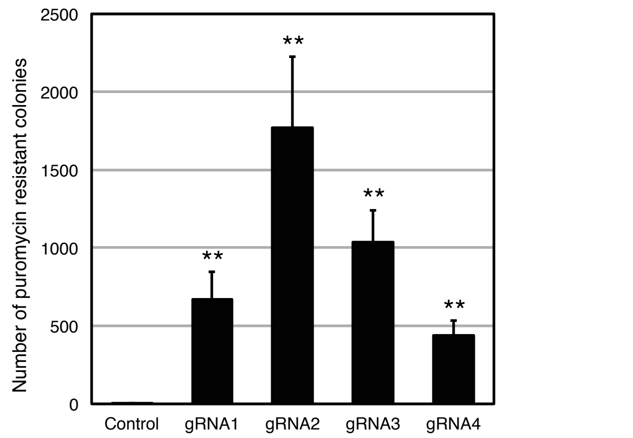

g)

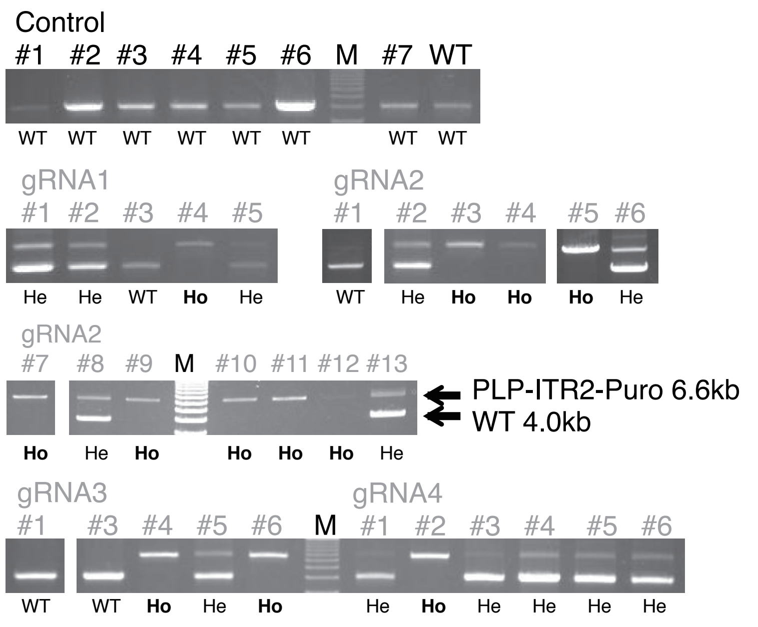

h)

Targeting Vector	Cas9-gRNA	Genotyped	KI clones (Homo-KI)	Targeting %
PLP-P216S	-/-	6	0 (0)	0%
PLP-P216S	+/PLP1-ITR5-1	12	12 (2)	100%
PLP-P216S	+/PLP1-ITR5-2	12	12 (8)	100%
PLP-P216S	+/PLP1-ITR5-3	12	12 (5)	100%
PLP-S253T	-/-	3	0 (0)	0%
PLP-S253T	+/PLP1-ITR5-2	6	6 (2)	100%
PLP-A39T	-/-	7	0 (0)	0%
PLP-A39T	+/PLP1-CDS2-1	5	4 (1)	80%
PLP-A39T	+/PLP1-CDS2-2	13	12 (8)	92%
PLP-A39T	+/PLP1-CDS2-3	5	3 (2)	60%
PLP-A39T	+/PLP1-CDS2-4	6	6 (1)	100%

Supplementary Figure S8

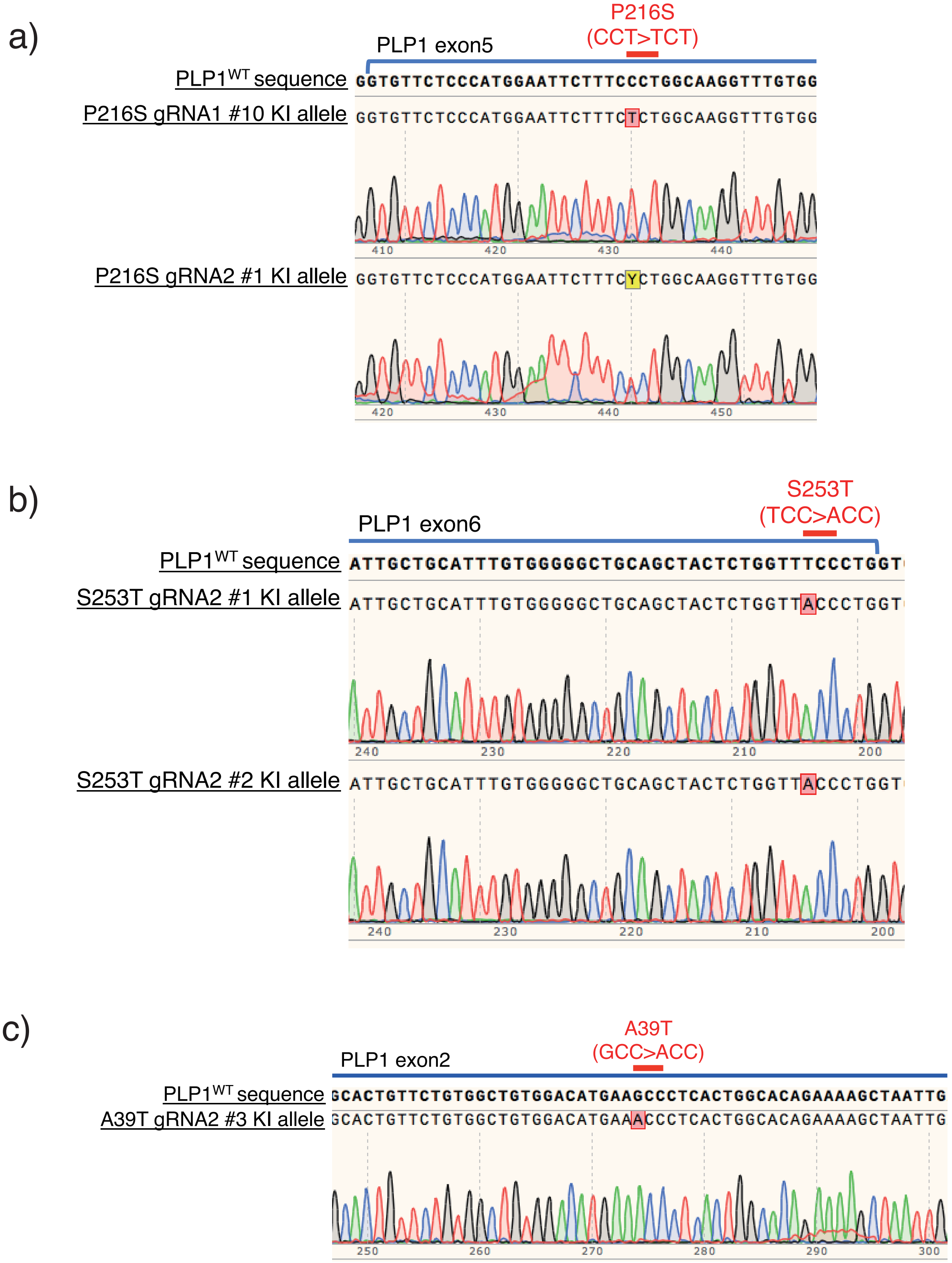

Supplementary Figure S9

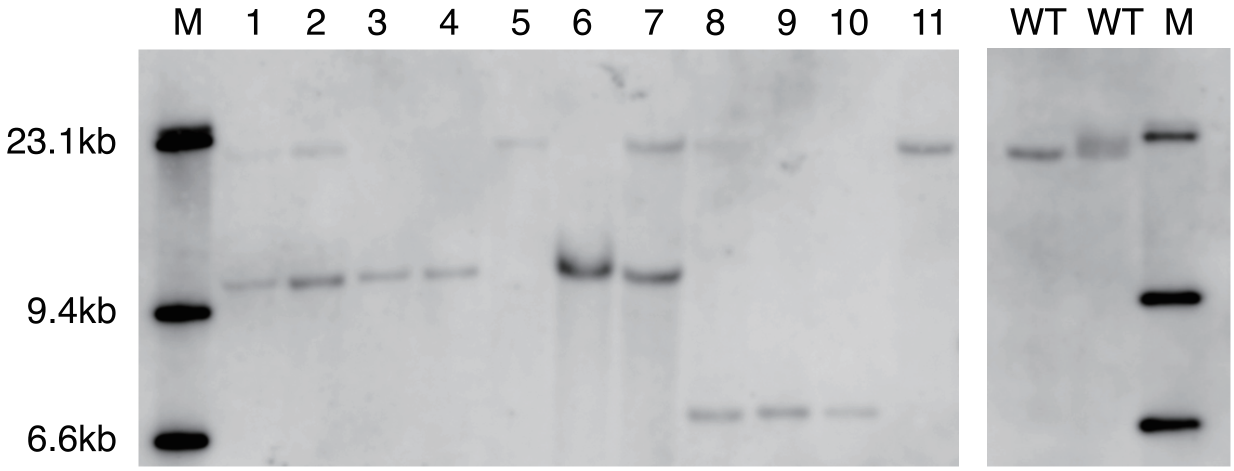

Lane No.	Targeting Vector	Cas9/gRNA	Clone No.	Genotype (PCR)	Genotype (Southern)
1	PLP1-P216S	+/ITR5-1	#2	KI/WT	KI/WT
2	PLP1-P216S	+/ITR5-1	#3	KI/WT	KI/WT
3	PLP1-P216S	+/ITR5-2	#1	KI/KI	KI/KI
4	PLP1-P216S	+/ITR5-3	#3	KI/KI	KI/KI
5	PLP1-P216S	-/-	#2	WT/WT	WT/WT
6	PLP1-S253T	+/ITR5-2	#1	KI/KI	KI/KI
7	PLP1-S253T	+/ITR5-2	#2	KI/WT	KI/WT
8	PLP1-A39T	+/CDS2-2	#2	KI/WT	KI/WT
9	PLP1-A39T	+/CDS2-2	#3	KI/KI	KI/KI
10	PLP1-A39T	+/CDS2-3	#6	KI/KI	KI/KI
11	PLP1-A39T	-/-	#6	WT/WT	WT/WT

Supplementary Figure S10

FOXP2 TV / Cas9+gRNA4
Genotyping PCR of puromycin-selected cjESC clones
(except #10-15 shown in Figure 3)

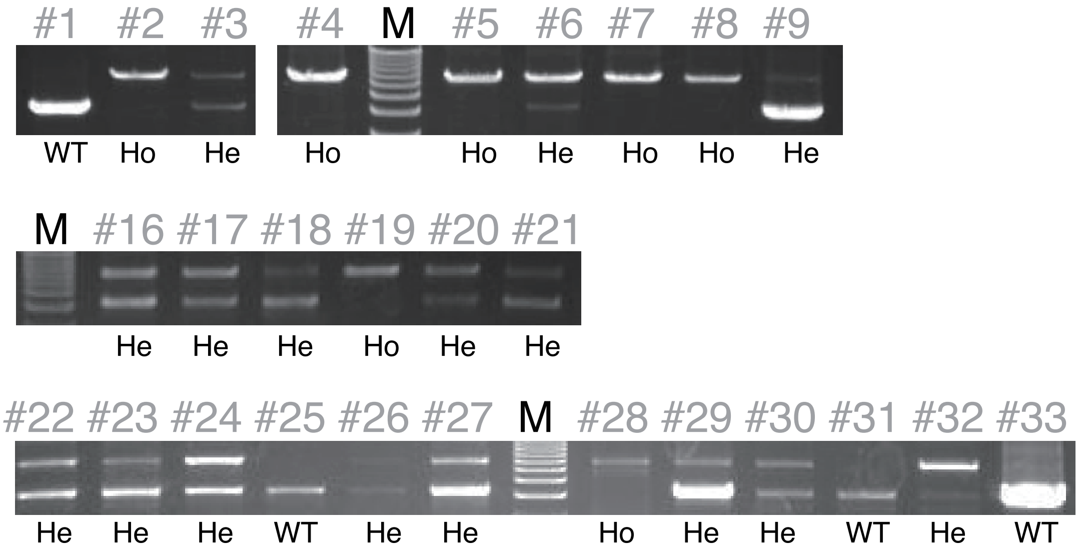

Supplementary Figure S11

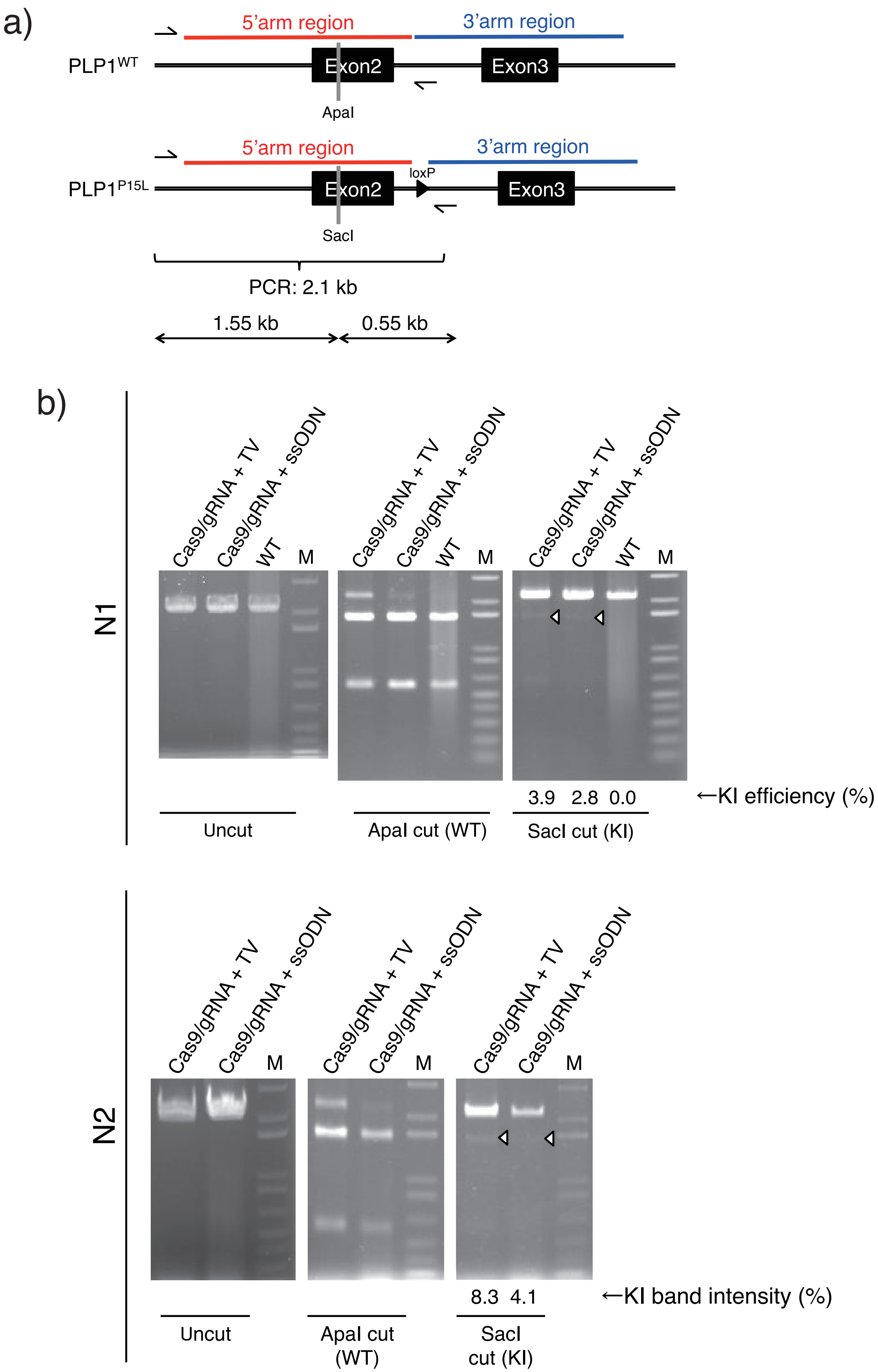

Supplementary Figure S12

a)

Condition	Used 2PNs	8-10 cells (Survival%)	PCR-Genotyped	WT	indel	KI	Modifying Efficiency %
Microinjection	38	18 (48.6%)	17	0	17	4	indel: 100%<br>KI: 24%

b)

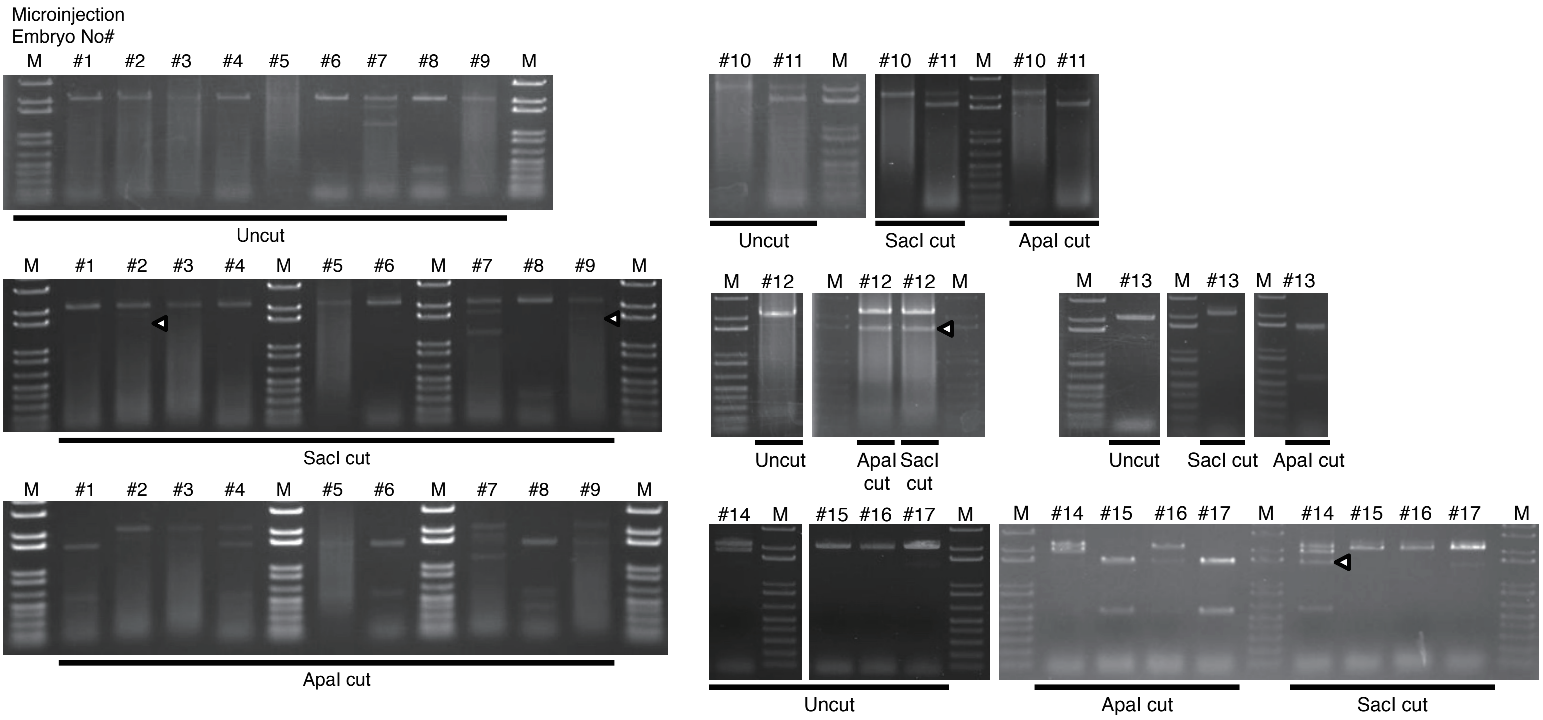

c)

```
                            gRNA        ApaI/SacI
WT allele : GCTGTGCAAGATGTCTGGTAGGGGCCCCCTTTGCTTCC
TV        : GTTGCGCTCGTTGCTTAGTTGGAGCTCTCTTTGCTTCC

Embryo #1
   +1bp (8/8): GCTGTGCAAGATGTCTG.TAGGGGCCCCCTTTGCTTCC

Embryo #2
     TVKI (4/9): GTTGCGCTCGTTGCTTAGTTGGAGCTCTCTTTGCTTCC
TVKI+loxP (2/9): GTTGCGCTCGTTGCTTAGTTGGAGCTCTCTTTGCTTCC +loxP
 -342/+1bp (2/9): GCTGTGCAAGATGG........................
    -13bp (1/9): GCTGTGCAA.A............GCCCCCTTTGCTTCC

Embryo #3
    -27bp (6/9): GCTGTGC...........................TTCC
   Intact (2/9): GCTGTGCAAGATGTCTGGTAGGGGCCCCCTTTGCTTCC
     -5bp (1/9): GCTGTGCAAGATGT.....AGGGGCCCCCTTTGCTTCC

Embryo #4
  -4/+6bp (5/7): GCTGTGCAAGATG....GTAGGGGCCCCCTTTGCTTCC
    -15bp (2/7): GCTGTGCAAGATGT...............TTTGCTTCC

Embryo #5
    -5bp (8/14): GCTGTGCAAGATGT.....AGGGGCCCCCTTTGCTTCC
  Intact (6/14): GCTGTGCAAGATGTCTGGTAGGGGCCCCCTTTGCTTCC

Embryo #6
    -10bp (5/7): GCTGTGCAA..........AGGGGCCCCCTTTGCTTCC
   Intact (2/7): GCTGTGCAAGATGTCTGGTAGGGGCCCCCTTTGCTTCC

Embryo #7
   Intact (6/7): GCTGTGCAAGATGTCTGGTAGGGGCCCCCTTTGCTTCC
    -27bp (1/7): GCT...........................TTGCTTCC

Embryo #8
   Intact (4/7): GCTGTGCAAGATGTCTGGTAGGGGCCCCCTTTGCTTCC
     -1bp (2/7): GCTGTGCAAGATGTCTG.TAGGGGCCCCCTTTGCTTCC
     +1bp (1/7): GCTGTGCAAGATGTCTGGTAGGGGCCCCCTTTGCTTCC

Embryo #9
TVKI+loxP (9/24): GTTGCGCTCGTTGCTTAGTTGGAGCTCTCTTTGCTTCC +loxP
     -3bp (9/24): GCTGTGCAAGATG...GGTAGGGGCCCCCTTTGCTTCC
   Intact (5/24): GCTGTGCAAGATGTCTGGTAGGGGCCCCCTTTGCTTCC
     TVKI (1/24): GTTGCGCTCGTTGCTTAGTTGGAGCTCTCTTTGCTTCC
```

```
                            gRNA        ApaI/SacI
WT allele : GCTGTGCAAGATGTCTGGTAGGGGCCCCCTTTGCTTCC
TV        : GTTGCGCTCGTTGCTTAGTTGGAGCTCTCTTTGCTTCC

Embryo #10
   -49bp (2/7): GCTGTGCAAGATGT........................
   -19bp (2/7): GCTGTGCAAGATGT..................GCTTCC
    -5bp (1/7): GCTGTGCAAGATGT.....AGGGGCCCCCTTTGCTTCC
  Intact (1/7): GCTGTGCAAGATGTCTGGTAGGGGCCCCCTTTGCTTCC
-3/+24bp (1/7): GCTGTGCAAGATGTCTG...GGGGCCCCCTTTGCTTCC
                                  ^
                     CACATCTTGCACAGCATTCTAACT
Embryo #11
 -415bp (13/16): GCTGTGCAAGATGTCT......................
  Intact (3/16): GCTGTGCAAGATGTCTGGTAGGGGCCCCCTTTGCTTCC

Embryo #12
     TVKI (12/18): GTTGCGCTCGTTGCTTAGTTGGAGCTCTCTTTGCTTCC
 TVKI+loxP (4/18): GTTGCGCTCGTTGCTTAGTTGGAGCTCTCTTTGCTTCC +loxP
    Intact (2/18): GCTGTGCAAGATGTCTGGTAGGGGCCCCCTTTGCTTCC
                                       G
Embryo #13                             v
     +1bp (9/10): GCTGTGCAAGATGTCTGGTAGGGGCCCCCTTTGCTTCC
   Intact (1/10): GCTGTGCAAGATGTCTGGTAGGGGCCCCCTTTGCTTCC

Embryo #14
   -191bp (6/10): GCTGTGCAAGATGTCT......................
 TVKI+loxP (4/10): GTTGCGCTCGTTGCTTAGTTGGAGCTCTCTTTGCTTCC +loxP

Embryo #15
  Intact   (6/11): GCTGTGCAAGATGTCTGGTAGGGGCCCCCTTTGCTTCC
     -1bp  (4/11): GCTGTGCAAGATGTCTG.TAGGGGCCCCCTTTGCTTCC
     -8bp  (1/11): GCTGTGCAAG........TAGGGGCCCCCTTTGCTTCC
                                     TTTT
Embryo #16                             v
-1bp/+4bp (7/10): GCTGTGCAAGATGTCTG.TAGGGGCCCCCTTTGCTTCC
    -11bp (3/10): GCTGTGCAAGATGTC...........CCCTTTGCTTCC

Embryo #17
     -1bp (6/10): GCTGTGCAAGATGTCTG.TAGGGGCCCCCTTTGCTTCC
-5bp/+4bp (4/10): GCTGTGCAAGATG.....TAGGGGCCCCCTTTGCTTCC
                                 ^
                                CAAA
```

Supplementary Figure S13

a)

Condition	Used 2PNs	8-10 cells (Survival%)	PCR-Genotyped	WT	indel	KI	Modifying Efficiency %
Electroporation	7	6 (86%)	6	2	4	2	indel: 67%<br>KI: 33%

b)

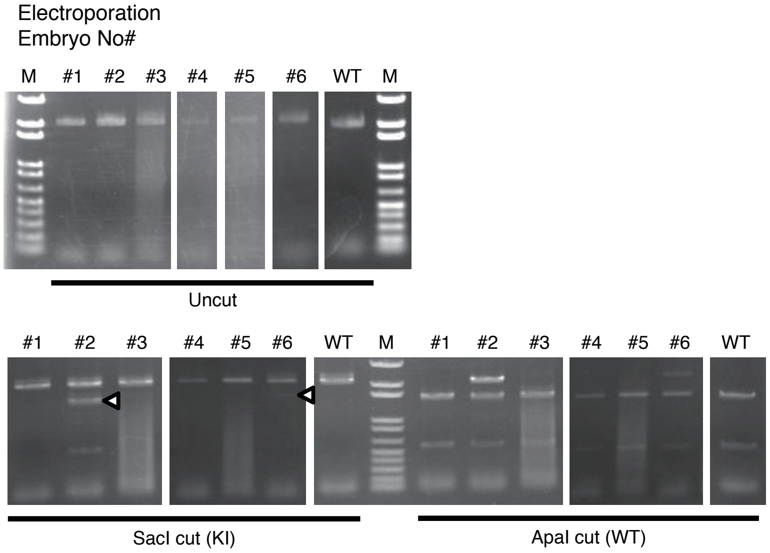

c) Electroporation
Embryo No#

```
                                   gRNA           ApaI/SacI
WT allele : GCTGTGCAAGATGTCTGGTAGGGGCCCCCTTTGCTTCC
ssODN     : GCTGTGCAAGATGTCTGGTTGGAGCTCTCTTCGCTTCC
TV        : GTTGCGCTCGTTGCTTAGTTGGAGCTCTCTTTGCTTCC

Embryo #1                            G
     +1bp (8/8): GCTGTGCAAGATGTCTGGTAGGGGCCCCCTTTGCTTCC

Embryo #2
ssODNKI  (9/16): GCTGTGCAAGATGTCTGGTTGGAGCTCTCTTCGCTTCC
    -1bp (7/16): GCTGTGCAAGATGTCTG.TAGGGGCCCCCTTTGCTTCC

                         ACTTGACTGGAAACA
Embryo #4
 -3/+15bp (1/6): GCTGTGCAAGATGT...GTAGGGGCCCCCTTTGCTTCC
     -1bp (5/6): GCTGTGCAAGATGTCTG.TAGGGGCCCCCTTTGCTTCC

Embryo #6
   TV KI (2/13): GTTGCGCTCGTTGCTTAGTTGGAGCTCTCTTTGCTTCC
    -1bp (7/13): GCTGTGCAAGATGTCTG.TAGGGGCCCCCTTTGCTTCC
      WT (4/13): GCTGTGCAAGATGTCTGGTAGGGGCCCCCTTTGCTTCC
```

Supplementary Figure S14

a)

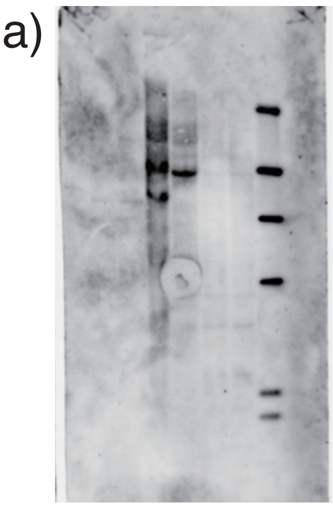

b)

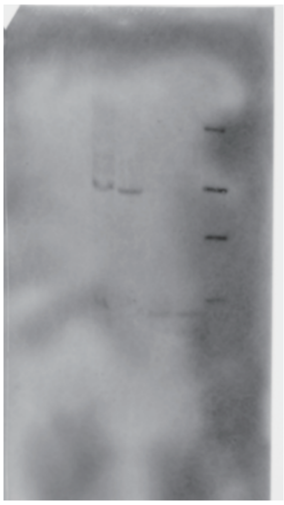

c)

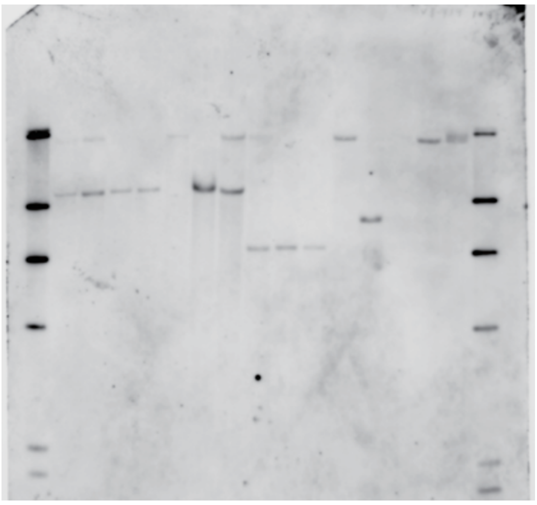

Supplement: Supplementary file 1 — Supplementary Information [file 41598_2018_37990_MOESM1_ESM.pdf]
